# Deficiency of leucine-rich repeat kinase 2 aggravates thioacetamide-induced acute liver failure and hepatic encephalopathy in mice

**DOI:** 10.1186/s12974-024-03125-4

**Published:** 2024-05-09

**Authors:** Dan Li, Shu-fang Yu, Lin Lin, Jie-ru Guo, Si-mei Huang, Xi-lin Wu, Han-lin You, Xiao-juan Cheng, Qiu-yang Zhang, Yu-qi Zeng, Xiao-dong Pan

**Affiliations:** 1 Department of Neurology, Fujian Institute of Geriatrics, Center for Cognitive Neurology, Fujian Medical University Union Hospital, 29 Xinquan Road, Fuzhou, 350001 China; 2 Department of Gastroenterology, Fujian Medical University Union Hospital, 29, Xinquan Road, Fujian, 350001 China; 3 Fujian Key Laboratory of Molecular Neurology, Fujian Medical University, 29 Xinquan Road, Fuzhou, 350001 China; 4 Fujian Key Laboratory of Vascular Aging, Fujian Medical University, Fuzhou, 350001 Fujian China; 5 Institute of Clinical Neurology, Fujian Medical University, 29 Xinquan Road, Fuzhou, 350001 China; 6 Fujian Clinical Research Center for Digestive System Tumors and Upper Gastrointestinal Diseases, Fujian, 350001 China; 7 Clinical Research Center for Precision Diagnosis and Treatment of Neurological Diseases of Fujian Province, Fuzhou, 350001 China

## Abstract

**Background:**

Hepatic encephalopathy (HE) is closely associated with inflammatory responses. However, as a crucial regulator of the immune and inflammatory responses, the role of leucine-rich repeat kinase 2 (LRRK2) in the pathogenesis of HE remains unraveled. Herein, we investigated this issue in thioacetamide (TAA)-induced HE following acute liver failure (ALF).

**Methods:**

TAA-induced HE mouse models of LRRK2 wild type (WT), LRRK2 G2019S mutation (*Lrrk2*^G2019S^) and LRRK2 knockout *(Lrrk2*^−/−^) were established. A battery of neurobehavioral experiments was conducted. The biochemical indexes and pro-inflammatory cytokines were detected. The prefrontal cortex (PFC), striatum (STR), hippocampus (HIP), and liver were examined by pathology and electron microscopy. The changes of autophagy-lysosomal pathway and activity of critical Rab GTPases were analyzed.

**Results:**

The *Lrrk2*^−/−^-HE model reported a significantly lower survival rate than the other two models (24% vs. 48%, respectively, *p* < 0.05), with no difference found between the WT-HE and *Lrrk2*^G2019S^-HE groups. Compared with the other groups, after the TAA injection, the *Lrrk2*^−/−^ group displayed a significant increase in ammonium and pro-inflammatory cytokines, aggravated hepatic inflammation/necrosis, decreased autophagy, and abnormal phosphorylation of lysosomal Rab10. All three models reported microglial activation, neuronal loss, disordered vesicle transmission, and damaged myelin structure. The *Lrrk2*^−/−^-HE mice presented no severer neuronal injury than the other genotypes.

**Conclusions:**

LRRK2 deficiency may exacerbate TAA-induced ALF and HE in mice, in which inflammatory response is evident in the brain and aggravated in the liver. These novel findings indicate a need of sufficient clinical awareness of the adverse effects of LRRK2 inhibitors on the liver.

**Supplementary Information:**

The online version contains supplementary material available at 10.1186/s12974-024-03125-4.

## Introduction

Hepatic encephalopathy (HE) is a neuropsychiatric abnormality in patients with acute or chronic liver diseases. These patients develop a progressive hypokinetic rigid syndrome that is termed as “hepatic parkinsonism or cirrhosis-related parkinsonism”, characterized by progressive ataxia, dystonia, and choreoathetosis accompanied by progressive cognitive dysfunction [1]. Noticeably, these parkinsonism-like pathophysiologic features can be manifested in HE, with alterations to the metabolism and transport of brain dopamine and to receptor integrity at basal ganglia. However, the exact HE pathogenesis remains obscure.

Of the potential mechanisms underlying HE, the notion of inflammation-fired neurotoxicity has recently received growing attention, despite the well-established ammoniacal poisoning hypothesis. Mounting evidence highlights the role of microglia in the occurrence and development of HE-induced central inflammation [2–5]. Studies have documented, in HE patients and rodent models with liver failure, the activation of microglia and hepatic macrophage, together with increases in pro-inflammatory factors, including tumor necrosis factor alpha (TNF-α), interleukin-1 beta (IL-1β), and interleukin-6 (IL-6) [2, 6, 7]. Moreover, Kupffer cells, specialized liver-resident macrophages, have been found to play an important role in acute liver failure (ALF) [8]. However, the co-mechanism underlying the activation of microglia and hepatic macrophages in HE patients remains largely unelucidated.

The available literature demonstrates that the homeostasis of several tissue-resident macrophage populations, including microglia, is regulated by leucine-rich repeat kinase 2 (LRRK2) [9–11] and that the mutations of LRRK2 are the commonest genetic cause of Parkinson’s disease (PD) [12–14], with G2019S mutation documented in sporadic PD [15]. To date, as a complex GTPase/kinase, LRRK2 orchestrates multiple steps of the endolysosomal pathway and autophagy-lysosomal pathway by interacting with a host of partners and phosphorylating a subset of Rab GTPases [16, 17]. Other studies have demonstrated that autophagy is implicated in various pathological processes, including HE [18], and that modulating autophagy in astrocytes and/or neurons may represent a novel treatment for liver diseases-associated HE [19]. Altogether, these findings signify a close involvement of LRRK2 in the HE pathogenesis.

The role of LRRK2 has been explored in various diseases. In inflammatory neurodegenerative disorders, several studies of the central nervous system (CNS) have reported the implication of LRRK2 in various inflammatory modulators such as mitogen-activated protein kinase (MAPK), nuclear factor of activated T1 cells 1 (NFAT1), nuclear factor kappa-B (NF-κB), and regulator of calcineurin 1 (RCAN1), and its regulation of diverse inflammatory pathways [20–26]. Cumulative evidence also suggests that the abnormal activity or mutations of LRRK2 might prompt the production of pro-inflammatory microglia and that an increase of microglial LRRK2 may promote α-synuclein uptake and clearance [27–30]. Furthermore, some studies have documented an interestingly opposing effect of LRRK2 in CNS and peripheral innate immunity [12]. Still some researchers have investigated the association of LRRK2 expression with the inflammation in lung and kidney. However, so far, few studies have probed into the role of LRRK2 in liver inflammation, let alone its role in HE.

In the current study, the role of LRRK2 in the maintenance of liver-brain integrity was investigated in a TAA-induced HE mouse model. We found that LRRK2 deficiency aggravated the hepatic inflammation of the LRRK2 knockout mice and partially exacerbated the neuroinflammation. This novel finding signifies a need of sufficient caution in clinical administration of LRRK2 inhibitors.

## Methods

### Mice

B6.129X1 (FVB)-LRRK2^tm1.1^Cai/J (stock No. 012453) and C57BL/6J-Tg (LRRK2*G2019S)2AMjff/J (stock No. 018715) mice were purchased from the Jackson Laboratory (Bar Harbor, Maine, USA). Age-matched, nontransgenic littermates were used as wild-type controls for all experiments. All animal procedures and experiments observed the National Guidance for Animal Experiment and were approved by the Animal Ethics Committee of Fujian Medical University (No. FJMU IACUC 2021-0313). The mice were allowed free access to food and water and housed in a standard facility maintained at 22–25 °C on a 12 h/12 h light/dark cycle.

### Induction of acute hepatic failure

**Fig. 1:**
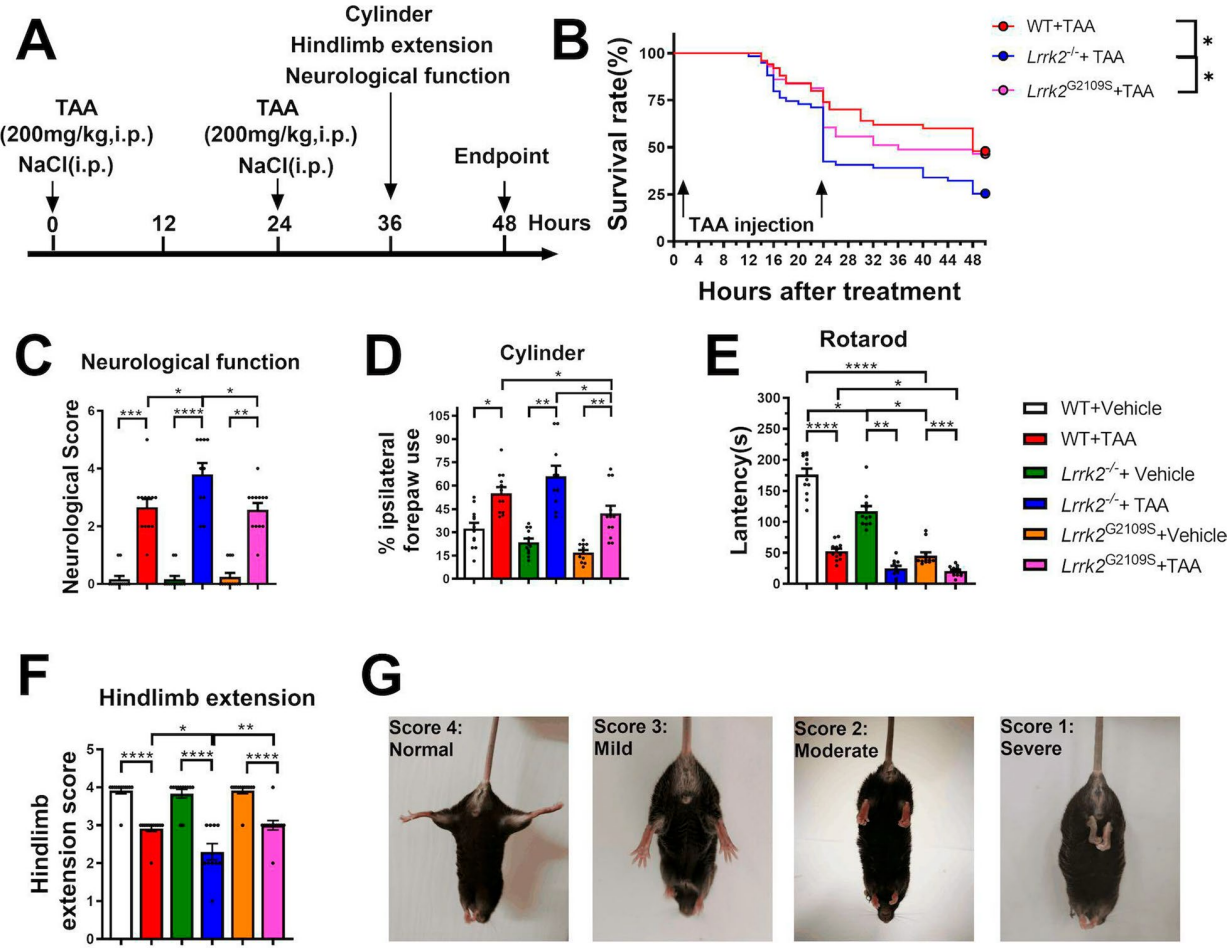
Survival and behavioral evaluation of mice. **A** A flow chart for TAA-induced HE mice model. **B** Survival rate of three TAA-intervened groups (n = 45–50). **C** Neurological score of each group, with the score of normal mice set as 0 (n = 10–12). **D** Cylinder test of each group, with the ordinate showing the percentage of unilateral front paw in the total front paw use (n = 10–12). **E** Residence time on the rod in the rotarod test in each group (n = 10–12). **F** The vertical coordinate shows the score of the mice in the hindlimb extension test, with that of the normal mouse set at 4 points (n = 10–12). **G** The score corresponding to the hindlimb extension of mice in the hindlimb extension test. For neurological score, cylinder test, rotarod test and hindlimb extension test, data were reported as mean ± SEM. *p < 0.05; **p < 0.01; ***p < 0.001; ****p < 0.0001, by Kruskal–Wallis test with Dunn’s comparison

Three genotypes of mice (aged 6–7 months) were respectively randomized into the treatment group and control group (45–50 mice/group). The ALF/HE model was established by two doses of single intraperitoneal injection of TAA (200 mg/kg; dissolved in 0.9% sterile saline beforehand) at an interval of 24 h. Control groups were injected with 0.9% sterile saline under the same conditions. To prevent hypovolemia, hypokalemia, and hypoglycemia, mice received a solution of 0.45% NaCl, 5% dextrose, and 0.2% KCl as a substitute for drinking water. The survival rate of each group was observed every two hours. At 36 h after the first injection, the treatment group underwent the neurological function test, rotarod test, cylinder experiment, and hindlimb extension test. Mice were anesthetized with a 1.5% ketamine solution and sacrificed at 48 h after the first injection. The liver, blood, and brain samples were subsequently collected for further analysis. The flow chart of the experiment is indicated in Fig. 1A.

## Behavioral tests

### Neurological function test

The neurological function of mice was assessed with a modified 10-point score scale evaluating the reflexes and task performance [31]. The former involved startle reflex, grasping reflex, righting reflex, placing reflex, corneal reflex; the latter included escaping from a circle (1 m in diameter) in less than 1 min, seeking, walking in a straight line, maintaining balance on a beam (1 cm in width), climbing onto a square and a round pole. For an abnormal reflex reaction or for each failed task, 1 point was assigned. Thus, with the brain function score of normal mice set as 0, a higher score indicated a poorer neurological function. The neurological function in each group was assessed at 36 h after the first TAA injection.

### Rotarod test

This method was similar to a previous study [32]. Mice were assessed for locomotor coordination and balance using a rotary fatigue apparatus (SA102, Jiangsu Sans biological technology Co., Ltd.). Before the test, the mice were placed and trained twice on a rotating rod (30 mm in diameter), which accelerated from 0 to 10 rpm. The training lasted for 15 min, with an interval of 30 m between the training. Twenty-four hours after the last training, mice underwent three trials at an interval of 30 m in between, with the rotating rod accelerating from 0 to 40 rpm and lasting for 5 min. The trial was completed when an animal fell off or reached a maximum duration. The time the mouse stayed on the rod in the three trials was recorded and averaged. A maximum of six mice were tested at once and the rotarod was cleaned after every session.

### Hindlimb extension test

As described previously [33], hindlimb extension test was performed to explore whether the mice suffered from dystonia. Mice were elevated by the tail by two independent investigators blinded to grouping details. The performance scores ranged from 1 to 4, with a lower score indicating severer dystonia. Score 4 was designated as “normal phenotype”, in which the mice reported no dystonia, with legs “V”-shaped widely from the body; score 3 as "mild phenotype", in which mice displayed no dystonia but were accompanied by trembling or failed to separate their legs from the body completely; score 2 as "moderate phenotype", in which mice showed symptoms of dystonia but were still able to move their legs; score 1 as "severe phenotype", in which mice failed to demonstrate any leg extensions or movement. The test score of each mouse was averaged from two trials, each lasting for 3–5 s.

### Cylinder test

The motor dysfunction was evaluated by a method adapted from a previous study [34]. The animals were placed individually inside a transparent cylinder (9 cm wide and 15 cm high) and observed for 5 min. The number of touching the cylinder wall with the left or right forelimb or both forelimbs was recorded by an operator blinded to the experimental groups. The calculation formula was as follows: (L + R)/(L + R + B) × 100% (L = left forelimb; R = right forelimb; B = both forelimbs), in which a value of = 50% indicates symmetric use of both forelimbs and that of > 50% signifies right or left forelimb preference.

## Serum liver enzyme and ammonia levels

Mouse blood sample was collected into lithium heparin anticoagulant tubes by eyeball enucleation. After 10 min on ice, the sample was centrifuged (3500 rpm, 6 min) and the upper serum was collected for analyses of aspartate aminotransferase (AST), alanine aminotransferase (ALT), and ammonia with a Vitros 5600 automatic biochemical analyzer (VITROS, USA).

## Liver and brain section preparation and histopathological staining

Mice were transcardially perfused with pre-cooled 0.9% sterile saline and subsequently with pre-cooled 4% paraformaldehyde. Liver and brain samples were isolated from six groups and post-fixed with 4% paraformaldehyde at 4 °C for 2–4 h. Before the preparation of frozen liver and brain sections, both samples were cryoprotected in 30% sucrose at 4 °C for 48 h, with the sucrose refreshed once after 24 h. Tissues were embedded in OCT compound (Sakura, CA, USA), sectioned (40 μm thick) with a freezing microtome (CM1950, Leica, Germany), and immersed in PBS at 4 ℃. The tissues were dehydrated with ethanol, embedded in paraffin, and subsequently sliced into sections (5 μm thick). The sliced sections were placed on poly-l-lysine-coated glass slides (ZSGB-BIO, Beijing, China).

### Liver H&E staining

H&E staining was performed to assess the morphology and necrosis of liver tissue, and the inflammatory extent. Briefly, hepatic sections were deparaffinized, rehydrated, and stained with Hematoxylin Staining Solution (ZSGB-BIO, Beijing, China) for 2 min. Subsequently, they were gradiently dehydrated in 75% and 95% ethanol (respectively for 5 min), stained with the eosin staining solution (ZSGB-BIO, Beijing, China) for 2 min, further gradiently dehydrated in 95% and 100% ethanol (respectively for 5 min), and finally transparentized with xylene twice (2 min per time). The sections were mounted onto coverslips with neutral gum. Cells were viewed under an optical microscope (Leica, Germany).

### Liver reticular fiber staining

To assess liver damage in mice, reticular fiber staining was performed on liver tissues. Briefly, liver paraffin sections were deparaffinized and dehydrated and subsequently treated with 1% potassium permanganate oxidation liquid (5 min), 2% oxalic acid (1 min), 2% ferric ammonium sulfate (1 min), ammonia silver solution (2 min), 10% formaldehyde solution (2 min), 0.2% gold chloride solution (1 min), and ponceau-picric acid (3 min), with a 5-min wash in distilled water between each step. Finally, the sections were dehydrated, cleared in xylene, and mounted with neutral gum. Staining was observed under an optical microscope (Leica, Germany), revealing a black hue of the reticular fibers.

### Brain Nissl staining

Brain sections from the prefrontal cortex, hippocampus (HIP), and striatum were respectively stained with Nissl staining solution (Beyotime, Shanghai, China) for 10 min following the manufacturer’s instructions. Nissl-positive cells were visualized under an optical microscope (Leica, Germany) and analyzed with Fiji software (Fiji, ImageJ 1.53c, NIH, Bethesda, MD, USA).

### Immunohistochemical staining

Liver sections were dewaxed with xylene, hydrated with gradient ethanol, and washed with phosphate buffer saline (0.01 M PBS). Antigen retrieval was performed by heating sections in a 0.01 M sodium citrate buffer (pH 6.0) or EDTA buffer (pH 8.0). After the treatment with endogenous peroxidase blocker for 20 min to block endogenous peroxidase activity, the sections were incubated with serum from the same source of secondary antibody at room temperature (RT) for 20 min to block nonspecific binding. Tissue sections were incubated overnight with LRRK2 antibody (1: 500, Abcam) and CD68 antibody (1: 500, Santa Cruz) at 4 °C before the introduction of secondary antibodies. Sections were further incubated in DAB to produce a brown reaction product, counterstained with hematoxylin, dehydrated, and transparentized. Similarly, brain sections received immunohistochemical staining with Iba1 antibody (1: 1000, WAKO), but without dewaxing, hydration, and antigen retrieval. Staining was observed under an optical microscope (Leica, Germany) and evaluated with Fiji software (Fiji, ImageJ 1.53c, NIH, Bethesda, MD, USA).

### Immunofluorescence staining

The expression of LAMP1 and Rab10 in liver tissues was determined by immunofluorescence staining. Briefly, the 40-μm liver sections were incubated in glycine solution at RT for 20 min after PBS washes. After the treatment with a blocking solution (0.3% Triton X-100, 1% BSA and 10% donkey serum) at RT for 1 h, the sections were incubated with primary antibodies (LAMP1, Rab10) at 4 °C overnight and subsequently with donkey anti-mouse IgG H + L (Alexa Fluor^®^ 488), donkey anti-rat IgG H + L (Alexa Fluor® 594) at RT for 1 h. To remove the autofluorescence in liver tissues, the sections were treated with a Vector True VIEW Autofluorescence Quenching kit (Vector, CA, USA) at RT in the dark for 2 min. Then, the nuclei were counterstained with DAPI (1: 10,000, Thermo Fisher).

The expression of TNF-α and IL-6 in PFC tissues was determined by immunofluorescence staining. The 40-μm brain sections were incubated in glycine solution at RT for 20 min after PBS washes. After the treatment with a blocking solution (0.3% Triton X-100, 1% BSA and 10% donkey serum) at RT for 1 h, the sections were incubated with primary antibodies (TNF-α, Proteintech; IL-6, CST) at 4 °C overnight and subsequently with donkey anti-mouse IgG H + L (Alexa Fluor^®^ 488) and donkey anti-rabbit IgG H + L (Alexa Fluor^®^ 594) for TNF-α, donkey anti-rabbit IgG H + L (Alexa Fluor^®^ 488) and donkey anti-goat IgG H + L (Alexa Fluor^®^ 594) for IL-6 at RT for 1 h. Then, the nuclei were counterstained with DAPI (1: 10,000, Thermo Fisher).

Finally, the anti-fluorescence quencher was used to seal the sections. The images were obtained by immunofluorescent microscopy (Observer A1, Zeiss, Germany) and photographed under an inverted fluorescence microscope (Leica, Germany).

### Image analysis and quantification of Nissl staining, Iba1 staining and immunofluorescence staining

After the relevant experiments, images were collected using identical image acquisition parameters and processed uniformly. For quantification, images were imported to Fiji software (Fiji, ImageJ 1.53c, NIH, Bethesda, MD, USA) and converted into 8-bit images. The percentage of the area occupied by cells was determined with the polygon tool; the thickness of different brain regions with straight-line tool; and counting with the cell counter tool. To analyze the number and morphology of neurons in the brain tissue, Nissl-stained cells were counted and the percentage of cells and thickness of different hippocampal regions were calculated from random visual fields. For quantification of microglial cells, Iba1 + cells were counted per high-power field (HPF). Sholl analyses were performed to quantify the degree of microglial activation. Images were processed by setting densitometric thresholds, smoothed, binarized, and skeletonized using the Skeletonize Plugin [35]. Templates of concentric circles were overlaid onto the center of a digitized cell soma, increasing the radius by a length of 3.0 μm to the end of the branch. The total number of over-threshold branches that intersected each circle was calculated and compared among groups. To detect the expression of specific markers in the liver tissues, the fluorescence intensity was quantified and expressed as mean fluorescence intensity (MFI). All images were processed and quantified by independent investigators blinded to the experimental condition.

## Western blotting

The liver tissues were lysed for 30 min in a RIPA buffer (Beyotime, Shanghai, China) containing a phosphatase inhibitor and a protease inhibitor (Roche). Subsequently, the tissue lysates were centrifuged at 4 °C for 15 min at 14,000 rpm to collect the supernatant, which was quantified by the BCA method (Beyotime, Shanghai, China). Protein extract (40 μg/well) was loaded into the ten wells in the 12% and 8% SDS–polyacrylamide gel (SDS-PAGE gel) and transferred to nitrocellulose membranes (NC membranes). Bands were blocked with 5% skim milk/TBST for 2 h and incubated in primary antibodies at 4 °C overnight, and further incubated in secondary antibodies at RT overnight. Bands were visualized with Bio-Rad ChemiDoc Imaging System using BeyoECL Star (Beyotime, Shanghai, China). Densitometric analysis was performed with Fiji software (Fiji, ImageJ 1.53c, NIH, Bethesda, MD, USA) and the protein band intensity was calculated by normalizing against the GAPDH band intensity. LC3B-II/LC3B-I ratio was calculated to evaluate the autophagy level of liver tissues in each group.

## RNA isolation and RT-qPCR

RNA was extracted from the liver tissues with a TRIzol Reagent (Invitrogen). cDNA synthesis was performed using a Revert Aid First Strand cDNA Synthesis Kit following the manufacturer’s instructions (Thermo Science, Waltham, USA). The quantitative RT-PCR (RT-qPCR) amplification was performed using FastStart Universal SYBR Green Master (ROX) (Roche) on the 7500 Real-Time PCR system (Applied Biosystems, South San Francisco, CA, USA). GAPDH mRNA levels were used as an internal normalization control. The specific primers are specified in Supplementary Table 1.

## TEM specimen preparation, image analysis, and quantification

Liver, prefrontal cortex, and striatum were quickly isolated from anaesthetized mice. Tissue blocks (1 mm^3^) were cut immediately and fixed with 3% glutaraldehyde-1.5% paraformaldehyde at 4 °C for 24 h; the tissues were post-fixed with 1% osmium acid-1.5% potassium ferrocyanide at RT for 1.5 h; they were then rinsed with PBS, stained with 70% uranium acetate saturated alcohol solution, dehydrated with alcohol-acetone, and embedded in epoxy resin (618 embedding medium). The ultrathin sections (90 nm) were stained with uranyl acetate and lead citrate for 5–15 min, and photographed under a TECNA1 transmission electron microscope (FEI, TECNAI G2). The myelinated and unmyelinated fibers were counted to calculate the percentage of myelinated fibers. The G-ratio of myelinated fibers was calculated as the ratio of the axonal diameter to the diameter of the myelinated fiber with Fiji software (Fiji, ImageJ 1.53c, NIH, Bethesda, MD, USA). The myelinated fibers shorter than 1 µm were referred to as the small myelinated fibers.

## Liver lysosome isolation

Crude lysosomal fractions were isolated from the liver tissues using a Lysosome Isolation Kit (No. LYSISO1, Sigma-Aldrich) following the manufacturer’s instructions. Briefly, an appropriate amount of liver tissues was weighed and washed with PBS and subsequently minced with 1X Extraction. The homogenate was centrifuged at 1000*g* for 10 min at 4 ℃ to remove large particles. The above steps were repeated and the supernatants were collected and centrifuged at 20,000*g* for 20 min. The pellets were saved and resuspended in 1X Extraction to obtain crude lysosomal fractions.

## Enzyme linked immunosorbent assay

All reagents were prepared following the instructions of the assay kits (EK282HS, EK206HS, EK296, EK201BHS, EK280HS, Liankebio). The brain tissues samples and diluted serum were added into the plates before a 90-min incubation. After washing, the plates received antibody, streptavidin labeled with horseradish peroxidase, enhancement solution, and streptavidin labeled with horseradish peroxidase, in that order, and respectively incubated for 30 min, 30 min, 15 min, and 15 min. Plates were washed each time before addition. The tetramethyl benzidine was incubated in the dark for 5–30 min. The stop solution was added into the plates. Lastly, the optical density was detected at 450 nm and 570 nm. The quantification of the IL-1β, IL-6, IL-8, TNF-α, and IFN-γ concentrations was performed by calibration with a standard curve.

## Statistical analyses

Survival analysis was presented as Kaplan–Meier survival curves with log-rank statistics. Data were expressed as mean ± SEM. For homogeneous variance, the data were analyzed by one-way analysis of variance (ANOVA) with post hoc *Tukey* multiple comparison test; for nonhomogeneous variance, the data were compared by non-parametric *Kruskal–Wallis rank sum* test with post hoc *Dunn* multiple comparison test. All statistical analyses were performed with GraphPad Prism V.7.0 (GraphPad Prism Software, San Diego, California, USA). A value of *p* < 0.05 was considered statistically significant.

## Results

### LRRK2 deficiency exacerbates neurological dysfunction and increases mortality in TAA-HE mice

Mouse models of TAA-HE were established to study the role of LRRK2 (Fig. 1A). After the TAA intervention, the mice displayed an acute response: flagging spirit, sluggish and progressively-weakened response to external stimuli, clammy skin, yellowish urine, and impaired motor ability (Fig. 1B). The TAA-treated groups reported significant differences in survival rate: 48% in the WT-HE group, 46.5% in the *Lrrk2*^G2019S^-HE group, but only 25.4% in the *Lrrk2*^*−/−*^-HE group (Fig. 1B) (Pairwise comparison between groups shown in Supplementary Fig. 1), indicating that LRRK2 deficiency significantly increases the mortality of mice after TAA intervention.

The behavioral tests revealed that TAA intervention greatly increased the brain function score of mice, with *Lrrk2*^*−/−*^-HE mice scoring the highest (Fig. 1C). TAA-treated mice reported motor incoordination (Fig. 1D), motor fatigue (Fig. 1E), and dystonia (Fig. 1F, G). Interestingly, the mice of the two genotypes exhibited different behavioral performances after drug administration. The *Lrrk2*^*−/−*^-HE mice displayed the most serious impairment in the cylinder test and hindlimb extension test (*p* < 0.05), while *Lrrk2*^G2019S^-HE group performed the worst in the rotarod test (*p* < 0.05). Overall, the results indicate that LRRK2 deficit impacts the TAA-HE mice more adversely and exacerbates the survival of the TAA-HE model when compared with an aberrant LRRK2 overexpression.

### LRRK2 deficiency aggravates liver injury and neuronal loss by inducing peripheral and central inflammation in TAA-HE mice

**Fig. 2:**
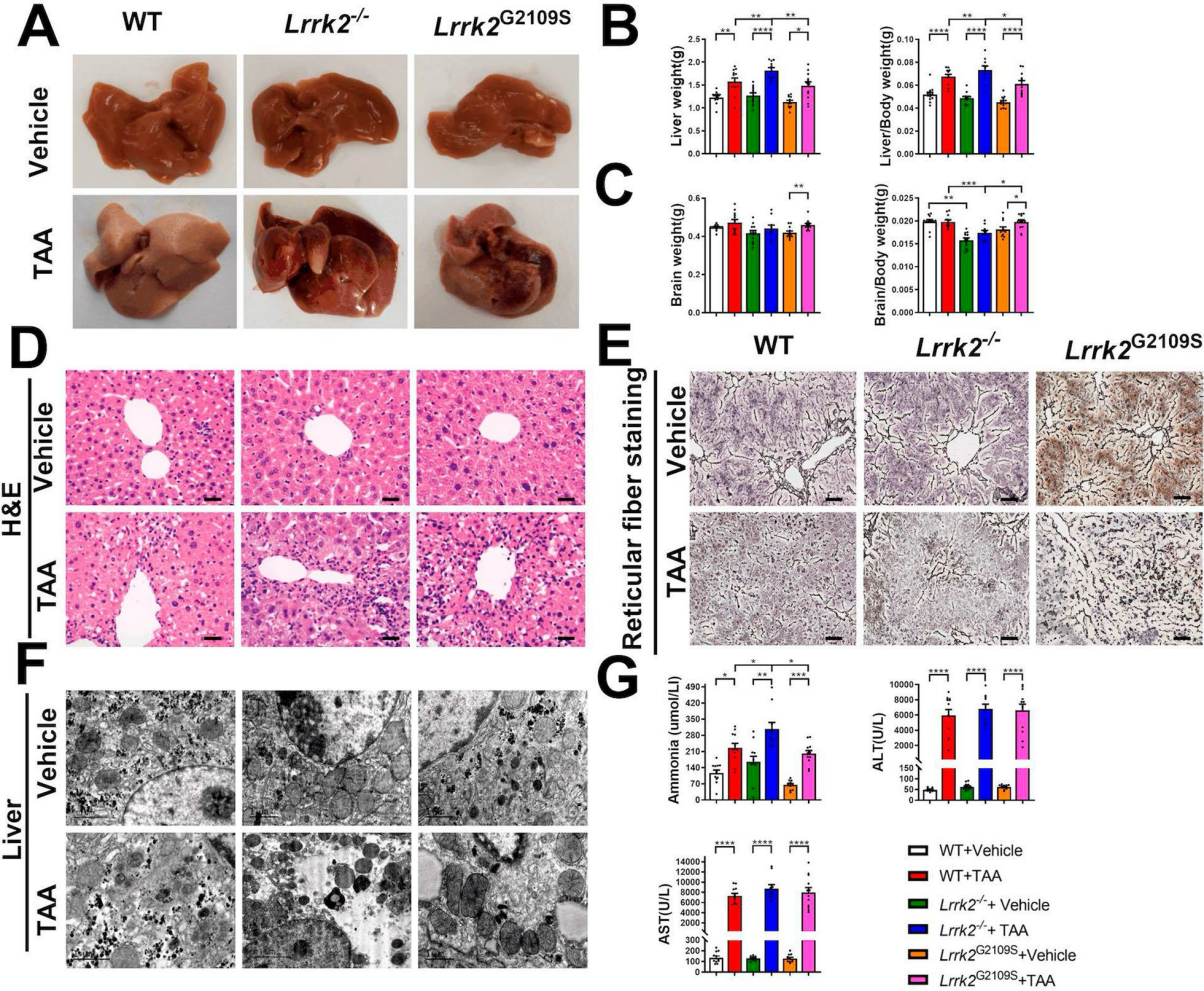
Pathological and physiological changes of liver. **A** General view of liver in each group. **B** Absolute and relative liver weight of each group (n = 10–12). **C** Absolute and relative brain tissue weight of each group (n = 10–12). **D** Pathological changes of liver tissue of each group by H&E histochemistry (Scale bars: 50 μm). **E** Reticular fiber changes of liver tissue of each group by reticular fiber staining (Scale bars: 50 μm). **F** Liver tissue of each group by transmission electron microscopy (Scale bars: 1 μm). **G** Levels of blood ammonia, ALT, and AST of each group (n = 10–12). For absolute liver weight, relative liver weight, absolute brain tissue weight, relative brain tissue weigh, ammonia, ALT and AST, data were reported as mean ± SEM. *p < 0.05; **p < 0.01; ***p < 0.001; ****p < 0.0001, by Kruskal–Wallis test with Dunn’s comparison

**Fig. 3:**
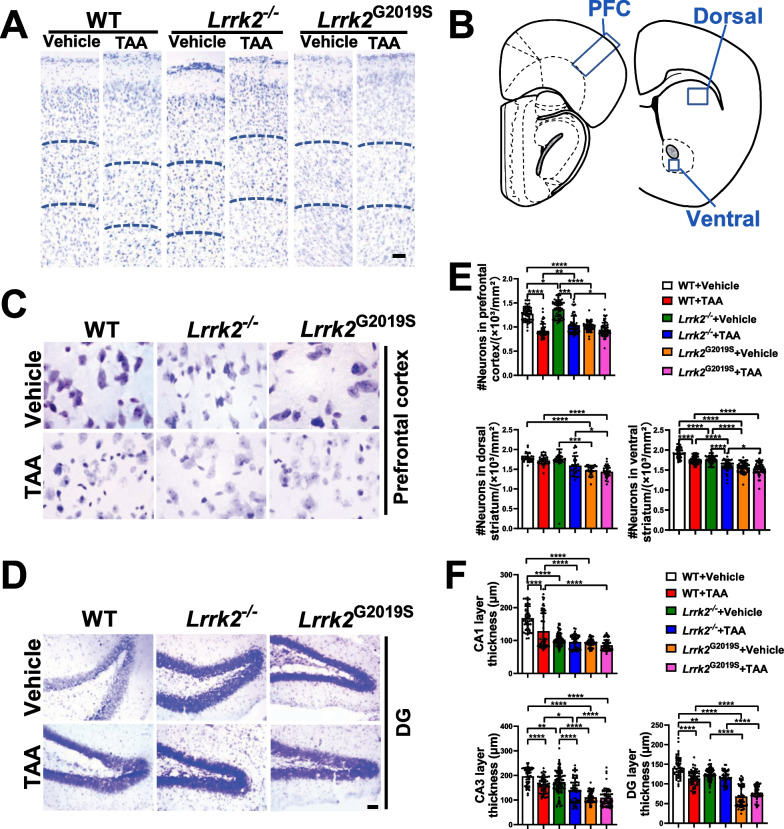
Representative images showing Nissl staining in brain tissues and quantification of damaged neurons. **A** Low-magnification images of the PFC (as shown in **B**) (Scale bars: 100 μm). Dashed lines indicate the borders of layer V. **C** High-magnification images of neuronal morphology in layer V of the PFC as indicated by area with dashed lines in **A** (Scale bars: 5 μm). **D** Low-magnification images of the hippocampal DG regions (Scale bars: 100 μm). **E** Quantification of the layer V neurons from the PFC, the dorsal and ventral STR (n = 6–8, 30–55 fields of views were observed for each mouse). Data are represented as mean ± SEM. and analyzed by one-way ANOVA test (*p < 0.05, **p < 0.01, ***p < 0.001, ****p < 0.0001). **F** Thickness of the cell layer in the hippocampal CA1, CA3 and DG (n = 6–8, 55–85 fields of views were observed for each mouse). Data are represented as mean ± SEM. and analyzed by one-way ANOVA test (*p < 0.05, **p < 0.01, ***p < 0.001, ****p < 0.0001)

The impact of LRRK2 on the function of important target organs was investigated by assessing pathological changes of liver and brain in the TAA-HE mice. Macroscopically, compared with the control group, the liver of the TAA-intervened mice featured general congestion, a swollen, dark red, rough surface, and extensive necrosis, especially in the *Lrrk2*^*−/−*^-HE mice (Fig. 2A), whereas no significant change was evident in the brain. The livers, brains and bodies of the mice were weighed and the proportion of liver and brain weight to that of the whole body was respectively calculated. The results showed that absolute and relative liver weight in TAA-intervened groups markedly increased (*p* < 0.05, respectively) (Fig. 2B). Notably, among all TAA-intervened groups, the absolute and relative liver weigh increased most significantly in the *Lrrk2*^*−/−*^-HE mice (*p* < 0.05); however, no significant difference was found in the absolute or relative weight of brain tissues (Fig. 2C). In H&E staining, compared with the control group, the TAA-intervened groups displayed hepatocytic edema and ballooning, reticular fiber collapse, infiltration of inflammatory cells (mainly macrophages and neutrophils), and punctate or focal necrosis of the hepatic lobules centered on the central vein, with the most significant inflammatory changes in the liver of the *Lrrk2*^*−/−*^-HE mice (Fig. 2D). Reticular fiber staining showed that the reticular fibers were complete in the control group, disconnected in the WT-HE and *Lrrk2*^G2019S^-HE groups, and collapsed in the *Lrrk2*^*−/−*^-HE group (Fig. 2E). The transmission electron microscopy revealed edema and steatosis of variable degrees in liver cells from the TAA-intervened mice, with the most serious hepatocytic necrosis in the *Lrrk2*^*−/−*^-HE mice (Fig. 2F). Nissl staining showed the pathological damage in brain tissues after the TAA treatment (Fig. 3A–D and Supplementary Fig. 2). Layer V neurons were consistently selected from the PFC, revealing swollen and fragmented neurons after the TAA intervention (Fig. 3C). Meanwhile, after the TAA treatment, the *Lrrk2*^*−/−*^ mice showed exacerbated partial neuronal loss, which was not the worst when compared with that of other TAA-intervened groups, while the *Lrrk2*^G2019S^ mice reported no significant changes in the PFC, STR and HIP before or after the TAA intervention (Fig. 3E, F).

Besides pathological manifestations, biochemical differences were evident in the liver. The TAA intervention caused serum hyperammonemia accompanied by significant increases in aspartate aminotransferase (AST) and alanine aminotransferase (ALT) (Fig. 2G). Likewise, the *Lrrk2*^*−/−*^-HE mice reported a higher level of blood ammonia (*p* < 0.05). The above results indicate that acute HE mice of different genotypes feature acute liver inflammation and necrosis to varying degrees, which can be exacerbated by LRRK2 deficiency, implying that LRRK2 plays a dual distinct role in hepatic inflammatory injury in HE mice.

### LRRK2 deficiency aggravates myelin damage and vesicle transport disorders in TAA-HE mice

**Fig. 4:**
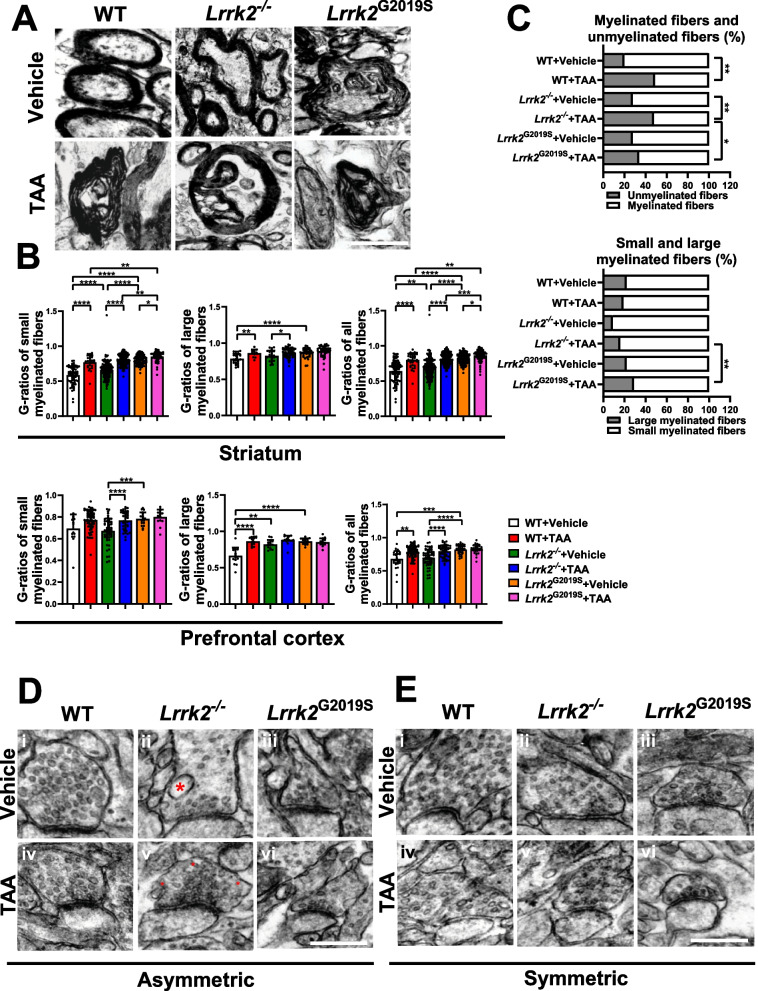
The morphological characterization of myelinated axons by electron microscopy in brain tissues and quantification of the regeneration and distribution of myelin sheaths. **A** Representative images of myelinated axons at STR by electron microscopy (Scale bars: 1 μm). **B** Quantification of myelin g-ratios at PFC and STR (n = 3, 5–10 fields of views were observed for each mouse). Data are represented as mean ± SEM and analyzed by one-way ANOVA test (*p < 0.05, **p < 0.01, ***p < 0.001, ****p < 0.0001). **C** Ratio of myelinated and unmyelinated fibers in STR (n = 3); ratio of large and small myelinated fibers in STR (n = 3) (Chi-square test; *p < 0.05, **p < 0.01). **D**, i–vi The images of vesicle at asymmetric synapses by electron microscopy (Scale bars: 500 μm). (ii) The vesicle of *Lrrk2*^*−/−*^ mice exhibited inconsistent size and sparse distribution, some with a significantly greater diameter (asterisk in ii). (v) The overall structural disorder in vesicle of the *Lrrk2*^*−/−*^-HE mice, including unusually formed endocytic intermediates (asterisk in v) and protein-coated vesicles with obscure borderline. **E**, i–vi The images of vesicle at symmetric synapses by electron microscopy (Scale bars: 500 μm). (i–iii) The sparser vesicle distribution in the *Lrrk2*^*−/−*^ mice (ii) than in other groups. (iv–vi) The abnormal vesicle shape in all TAA-HE groups

Next, we analyzed the nerve fibers by transmission electron microscopy. The results showed disrupted myelin sheath, hyperdense axoplasm, and vacuolization in the HE mice, particularly in the *Lrrk2*^G2019S^-HE mice (Fig. 4A). G-ratio analysis revealed that the TAA intervention led to demyelination and regeneration of neural fibers in the STR and PFC regions, thus resulting in an increased G-ratio (Fig. 4B). TAA intervention also reduced the proportion of myelinated neural fibers in the STR (Fig. 4C). After the TAA intervention, the differences observed were mainly evident in the STR, with a significantly higher overall fiber G-ratio in the *Lrrk2*^G2019S^-HE mice than in the WT-HE and *Lrrk2*^*−/−*^-HE groups. This phenomenon was particularly noticeable in the small myelinated fibers of the *Lrrk2*^G2019S^-HE mice (Fig. 4B), accompanied by a decrease in the proportion of small myelinated fibers (Fig. 4C). These results suggest that LRRK2 overexpression aggravates myelin damage in the TAA-HE mice.

The functional blockade of nerve conduction was further explored. The PFC brain sections of the mice were examined by transmission electron microscopy, reporting a similar number of asymmetric and symmetric synapses in each group (Fig. 4D, E). In asymmetric synapses, the vesicle size in the *Lrrk2*^*−/−*^mice was not consistent and distributed sparsely, with some vesicles exhibiting a significantly greater diameter (Fig. 4D, asterisk in ii), which was prevalent in all groups after the TAA injection (Fig. 4D, iv–vi); in symmetric synapses, the vesicles were more sparsely-distributed in the *Lrrk*2^*−/−*^ mice than in the other groups (Fig. 4E, i–iii) and all HE groups reported abnormal vesicle shape (Fig. 4E, iv–vi). Of note, in asymmetric synapses, the *Lrrk*2^*−/−*^-HE group exhibited more pronounced abnormalities in vesicle destruction than the WT-HE and *Lrrk2*^G2019S^-HE groups. Apart from the varied sizes, the vesicle of the *Lrrk*2^*−/−*^-HE mice reported an overall structural disorder, including unusually-formed endocytic intermediates (Fig. 4D, asterisk in v) and protein-coated vesicles with less density (Fig. 4D, v). These findings suggest that LRRK2 knockout aggravates the injury to the vesicle shape in the TAA-HE mice.

### Effect of LRRK2 knockout on the changes of acute phase cytokines in the liver and nervous system of TAA-HE mice

**Fig. 5:**
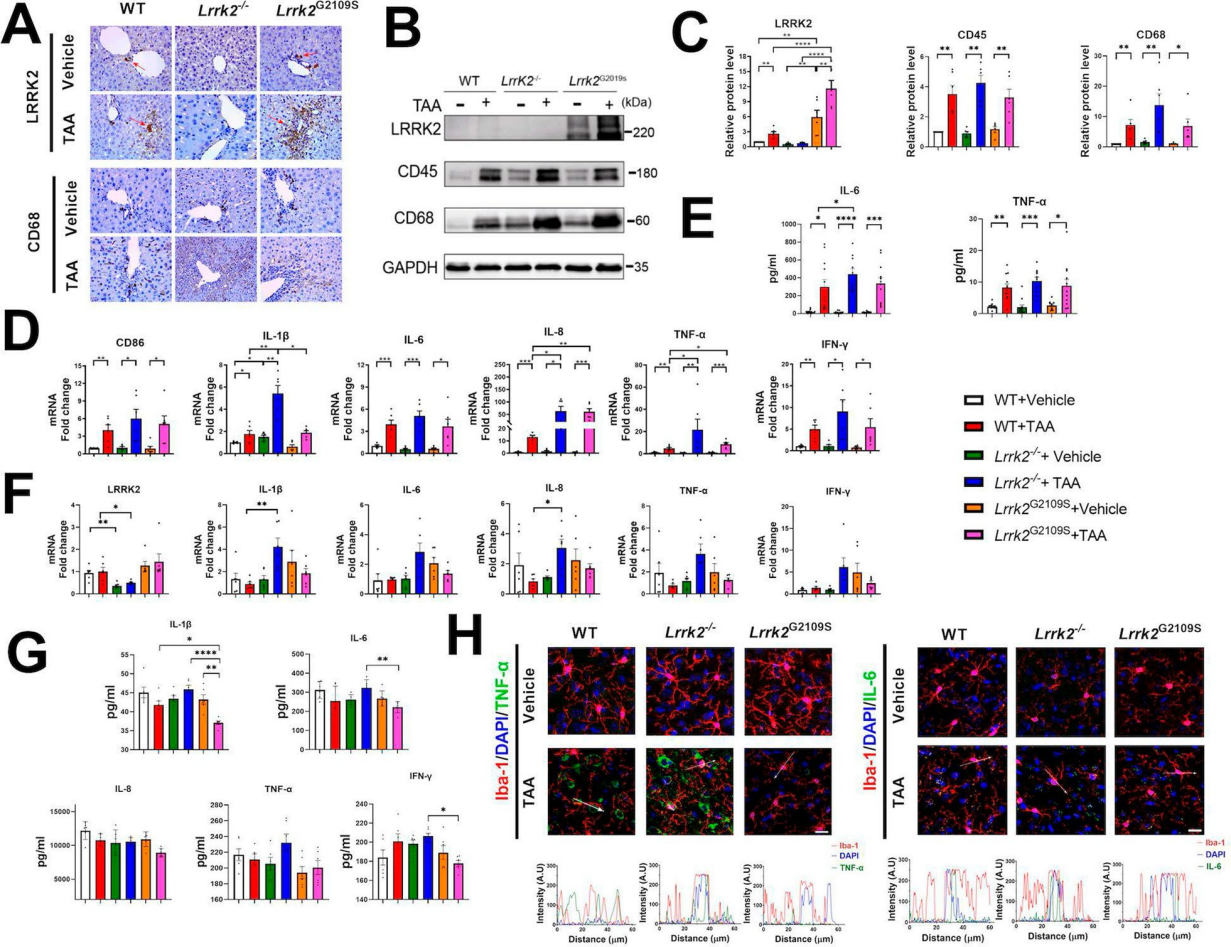
The expression of inflammatory factors in peripheral circulation, liver, and brain tissues of each group. **A** Liver proteins CD68 and LRRK2 expressions were assessed in each group by immunohistochemistry. **B** Liver total proteins LRRK2, CD68 and CD45 expression in each group (**C**) quantified by densitometry (n = 6). **D** The liver mRNA levels of CD86, IL-1β, IL-6, IL-8, TNF-α and IFN-γ, were assessed by qPCR (n = 6). **E** Peripheral IL-6 and TNF-α levels were detected by ELISA (n = 10–12). **F** LRRK2, IL-1β, IL-6, IL-8, TNF-α and IFN-γ mRNA expression in the PFC of each group were assessed by qPCR (n = 6). **G** IL-1β, IL-6, IL-8, TNF-α and IFN-γ levels in the PFC of each group were detected by ELISA (n = 6). **H** The colocalization of Iba1-expressing microglia (red) and TNF-α/IL-6 (green) in each group. DAPI (blue) was used as a nuclear stain (Scale bars: 25 μm). Representative fluorescent signal intensity plots (arrows in colocalization images) in each genotype after TAA intervention are indicated below the images. For protein, ELISA and mRNA analyses, data are reported as mean ± SEM. and analyzed by Kruskal–Wallis test with Dunn’s comparison. *p < 0.05; **p < 0.01; ***p < 0.001; ****p < 0.0001

We further explored the role of LRRK2 in the occurrence and development of acute liver inflammation. First, the expression of LRRK2 was verified in each group by western blotting and immunohistochemical staining. The result reported a significantly higher LRRK2 expression in the *Lrrk2*^G2019S^ mice than in the WT mice. After the TAA intervention, LRRK2 was upregulated in both the *Lrrk2*^G2019S^ mice and WT mice. CD68 and CD45, two molecular markers on the surface of macrophages, were detected by western blotting and immunohistochemical staining. Similarly, the expression of CD68 and CD45 increased after the TAA intervention, with the *Lrrk2*^*−/−*^-HE mice reporting the highest CD68, followed by the *Lrrk2*^G2019S^-HE mice, and then the WT-HE mice (*p* < 0.05) (Fig. 5A–C). These results suggest that after the TAA intervention, LRRK2 knockout promotes the activation and recruitment of macrophages in the liver, especially in the portal area of the *Lrrk2*^*−/−*^-HE mice.

Moreover, the mRNA levels of CD86 and acute inflammatory cytokines such as IL-1β, IL-6, IL-8, TNF-α, IFN-γ significantly increased in all TAA-intervened mice, especially in the *Lrrk2*^*−/−*^-HE mice (*p* < 0.05) (Fig. 5D and Supplementary Fig. 3A). In the plasma, ELISA analysis reported an upregulation of the inflammatory factors TNF-α and IL-6 in the TAA-intervened mice, especially in the *Lrrk2*^*−/−*^-HE mice (Fig. 5E). These results evidence the severest peripheral inflammation in the *Lrrk2*^*−/−*^-HE mice, consistent with the inflammatory response in the liver.

In the brain tissues of the *Lrrk2*^*−/−*^-HE mice, qPCR results showed that acute inflammatory cytokines such as IL-1β, IL-6, IL-8, TNF-α, IFN-γ were upregulated (Fig. 5F and Supplementary Fig. 3B), consistent with those of liver and peripheral circulation. Of note, the baseline values of some inflammatory factors in the *Lrrk2*^G2019S^ mice were higher than those of the other two control groups. After the TAA intervention, the proinflammatory cytokines declined in *the Lrrk2*^G2019S^ mice but no significant difference was found when the *Lrrk2*^G2019S^-HE mice were, respectively compared with its own control group and the WT group. Additionally, the ELISA analysis of the PFC tissue homogenates indicated that compared with the other genotype counterparts, *Lrrk2*^*−/−*^ mice reported a higher level of increased cytokines after the TAA intervention (Fig. 5G). In the *Lrrk2*^*−/−*^-HE mice, the immunofluorescence staining revealed that TNF-α and IL-6 were released from the microglia to the extracellular space and interacted with other cellular entities, including neurons with large nuclei (Fig. 5H). The above results indicate the involvement of LRRK2 in ALF and HE by promoting the activation and recruitment of macrophages to the liver and the release of inflammatory cytokines.

### LRRK2 deficiency increases microglial dysfunction in TAA-HE mice

**Fig. 6:**
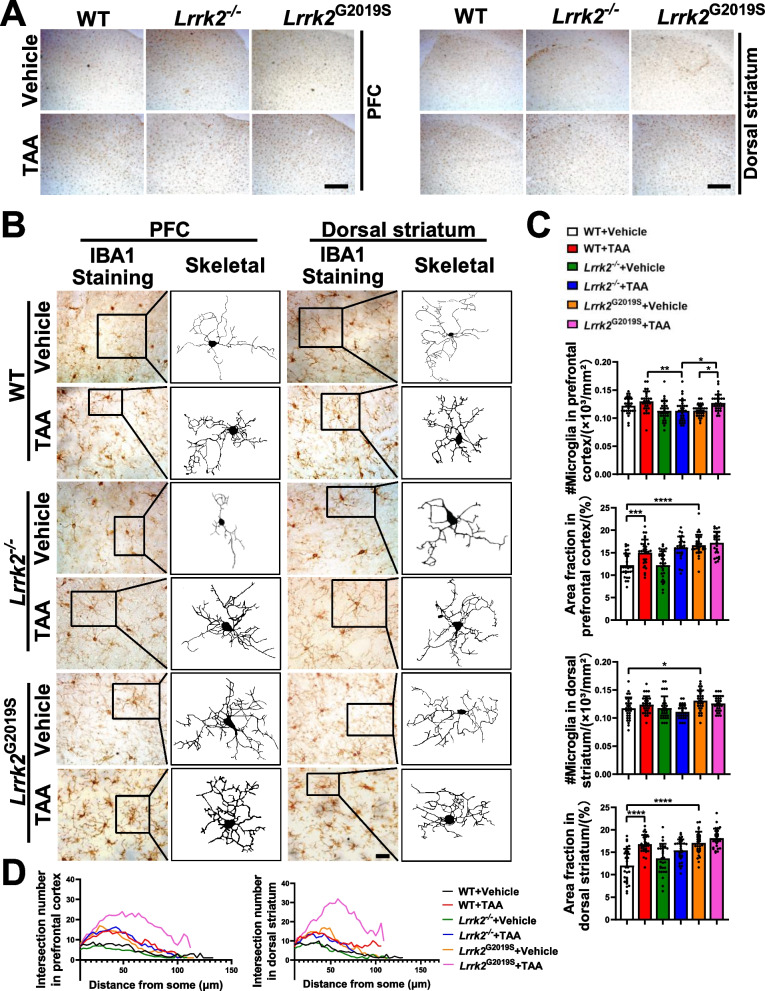
The representative images and quantification of Iba1-expressing microglia in brain tissues. **A** Low-magnification images of the PFC and dorsal STR (Scale bars: 500 μm). **B** High-magnification images of the PFC and the dorsal STR, with representative images of binarized microglial morphology (Scale bars: 50 μm). **C** Cell counts and calculation of area fraction of microglia in the PFC and dorsal STR (n = 6–8, 30 fields of views were observed for each mouse). Data are represented as mean ± SEM. and analyzed by one-way ANOVA test (*p < 0.05, **p < 0.01, ***p < 0.001, ****p < 0.0001). **D** Sholl analyses of microglia morphology (n = 6–8, 10 fields of views were observed for each mouse)

Given the role of microglial activation in HE progression, we next explored microglial activation in acute cerebral inflammation. With Iba1 as a general microglial marker, the microglial activity was detected by examining the microglial morphological changes in the dorsal STR and PFC (Fig. 6A). The results showed that after the TAA intervention, microglia were activated in all TAA-intervened mice, with the overall density and branch number of Iba1-positive microglia significantly increased, and microglial ramification retracted and thickened (Fig. 6B–D). Compared with the control groups, the *Lrrk2*^*−/−*^-HE and *Lrrk2*^G2019S^-HE mice reported no significant change in the fraction of the total area. However, the *Lrrk2*^G2019S^-HE mice reported retracted and thickened microglial ramifications in the Sholl analysis (Fig. 6B, D) and an increased number of microglia in the PFC (Fig. 6C). After the TAA intervention, the analysis of the phenotypes of microglia in each genotype revealed a significantly higher number of glial cells in the PFC of the *Lrrk2*^G2019S^-HE mice than in that of the *Lrrk2*^*−/−*^-HE group in terms of absolute cell count (Fig. 6C). Sholl analysis further showed that compared with the WT-HE counterparts, the *Lrrk2*^*−/−*^-HE mice exhibited a slightly denser and elongated branching of microglia in the PFC and STR. In contrast, the *Lrrk2*^G2019S^-HE mice indicated a contraction and thickening of glial cell branches (Fig. 6B, D). These results evidence that microglia exert distinct effects on the morphological changes in the *Lrrk2*^*−/−*^-HE and *Lrrk2*^G2019S^-HE mice.

### LRRK2 deficiency interferes with Rab10 phosphorylation and the autophagic-lysosomal pathway in TAA-HE mice

**Fig. 7:**
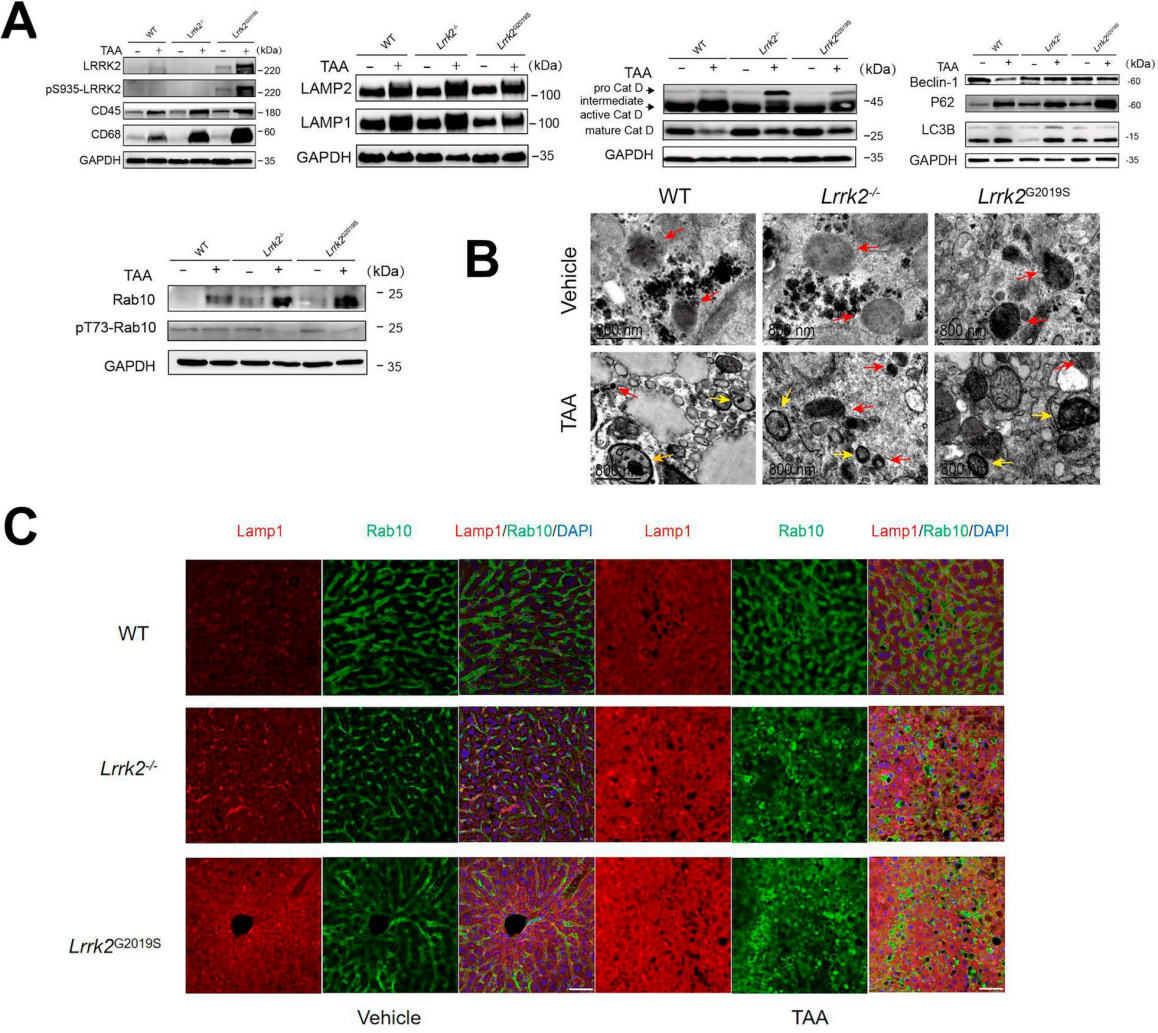
The expression of autophagy-related genes in each group. **A** Liver lysosomal proteins (LRRK2, CD45, CD68, LAMP1, LAMP2, Cath-D, Beclin1, LC3B, p62, and Rab10) in the liver tissues of each group were examined by western blotting (n = 6). **B** The lysosomes (red arrows), autophagosomes (yellow arrows) and autolysosome (orange arrows) of each group were shown by transmission electron microscopy (Scale bars: 800 nm). **C** The colocalization of Lamp1 (red) and Rab10 (green) in each group. DAPI (blue) was used as a nuclear stain (Scale bars: 50 μm). For protein analyses, data are reported as mean ± SEM. and analyzed by Kruskal–Wallis test with Dunn’s comparison. *p < 0.05; **p < 0.01; ***p < 0.001; ****p < 0.0001

Since autophagy plays an important role in the progress of inflammation caused by LRRK2-related diseases, we examined the expressions of three important autophagy-related proteins, Beclin 1, microtubule-associated protein 1 light chain 3 beta (LC3B), and p62 to investigate the changes of autophagy in mice undergoing acute inflammation after the TAA intervention. Western blotting revealed significantly up-regulated protein levels of LC3B I-converted LC3B II and p62 in the HE mice when compared with the controls, despite no significant change in Beclin 1 protein level (Fig. 7A and Supplementary Fig. 4). This finding suggests that despite the initial activation of autophagy of hepatocytes during the acute inflammation, autophagy flow is obstructed in the TAA-HE model and fails to maintain the homeostasis of the internal environment.

Lysosome is the end point of numerous vesicle trafficking pathways, including autophagic and endocytic lysosomal pathways. Western blotting was performed to detect the levels of the lysosome-associated membrane proteins 1 (LAMP1) and 2 (LAMP2), and the soluble lysosomal enzyme Cathepsin D (Cath-D), the latter acting as an indirect marker enzyme to evaluate the lysosomal function. In the acidic milieu of lysosomes, pro-Cath-D can be processed into intermediate active Cath-D and mature Cath-D. The results reported a markedly increase in LAMP1, LAMP2, and pro-Cath-D, but a decrease in intermediate active Cath-D and mature Cath-D in the TAA-HE mice (Fig. 7A and Supplementary Fig. 4). Compared with the WT and *Lrrk2*^G2019S^ mice, the *Lrrk2*^*−/−*^ -HE mice reported the highest expression of pro-Cath-D. These findings suggest that LRRK2 deficiency aggravates the TAA-induced blockage of autophagic-lysosomal pathway during the acute inflammatory response. The electron microscopy displayed the changes in the number of secondary lysosomes and autophagolysosomes in the liver after the TAA intervention, as well as the swelling and degeneration of cells and organelles (Fig. 7B). In the *Lrrk2*^*−/−*^-HE mice, an increase in autophagosomal formation and lysosomal destruction was observed. These results suggest that LRRK2 deficiency impairs the progress of autophagic-lysosomal pathway in the liver during the acute inflammatory response.

As LRRK2 can be recruited to stressed lysosomes, where it recruits and phosphorylates its substrate, small Rab GTPases, to maintain lysosomal homeostasis [36], we next investigated if LRRK2 affects lysosomal homeostasis through Rab protein in the TAA-HE model. Lysosomal proteins were purified with a lysosome isolation kit. The results showed that after the TAA intervention no significant difference was found in the expression of Rab5, Rab7, Rab8 and Rab29 in the liver lysosomes among groups (Supplementary Fig. 5) and that the total Rab10 was increased in all TAA-intervened mice and significantly upregulated in the *Lrrk2*^G2019S^-HE mice and *Lrrk2*^*−/−*^-HE mice (Fig. 7A). Subcellular localization of Rab10 and LAMP1 was detected by immunofluorescence (Fig. 7C). As expected, the WT and *Lrrk2*^G2019S^ mice, after the TAA intervention, reported an increased protein level of Rab10 and enhanced respective colocalization with lysosomes, while the *Lrrk2*^*−/−*^ mice reported an increase in Rab10 protein level and an increased colocalization of LAMP1 with lysosomes. As Rab10 can be phosphorylated at Thr73 by LRRK2, the phosphorylation of Rab10 was detected, which was lower in the *Lrrk2*^*−/−*^-HE mice than in the WT-HE or *Lrrk2*^G2019S^-HE mice (Fig. 7A and Supplementary Fig. 4). These results suggest that in the TAA-HE mice, LRRK2 may interfere with the lysosomal homeostasis by regulating Rab10 phosphorylation, thereby affecting autophagy.

## Discussion

Currently, the pathogenesis of HE, an important complication of end-stage liver disease, is poorly elucidated and much remains to be desired, despite the intense research into the role of hyperammonemia in its pathogenesis. Recently, the notion of the systemic inflammation theory has attracted growing attention, which hypothesizes that the inflammatory response may promote the occurrence and pathophysiological progression of HE.

The current study capitalized on the strong association between LRRK2 and systemic inflammation and explored the pathogenesis of TAA-induced ALF and HE. Our data first revealed that in TAA-induced mouse models of ALF and HE, the survival rate was twofold higher in the control group than in the *Lrrk2*^−/−^-mice and that LRRK2 deficiency significantly aggravated TAA-induced acute hepatic inflammation, which featured, to varying degrees, large focal hepatocytic necrosis, abnormal morphology in residual hepatocytes, edema, hepatocytic degeneration, cholestasis, collapse of reticular fiber scaffolds, infiltration of lobular inflammatory cells, and significant increase of acute inflammatory factors in tissues and peripheral circulation. Further mechanistic research reported that LRRK2 knockout affected the autophagy-regulated cell homeostasis to protect against inflammation by interfering with hepatic autophagic-lysosomal signaling pathway. However, in the central nervous system, despite the disrupted synaptic vesicle trafficking, LRRK2 deletion, to some extent, attenuated the myelination and neuronal loss via microglial activation-mediated central inflammation. These findings suggest that LRRK2 affects the central and peripheral systems in distinct inflammatory regulatory mechanisms. This discrepancy indicates a potential immune exemption and protection mechanism in the central nervous system, signifying that LRRK2 may fundamentally regulate the systemic immune inflammation in the TAA-HE model through peripheral regulation and that the observed high mortality rate of the model may be attributed to peripheral rather than cerebral causes.

The role of LRRK2 in the pathogenesis of PD has been clarified, but its role in systemic inflammatory diseases is far from clarification. It is well documented that LRRK2 is highly expressed in macrophages, monocytes, and neutrophils, strongly implicating its potential role as a regulator of immune responses [37]. Genomewide association studies have identified LRRK2 as a risk factor for peripheral inflammatory responses in leprosy infection [38], bacterial infection and inflammatory bowel disease (IBD) [39]. The differences in the outcome of LRRK2-regulated inflammation may be related to different types of LRRK2-expressing immune cells and the signaling pathways involved. LRRK2 has also been found to negatively regulate NFAT and reduce the susceptibility to DSS-induced IBD, indicating that LRRK2 may inhibit the progression of inflammation [29].

Currently, most studies of the role of LRRK2 in immunity and inflammation involve organs with abundant expression of LRRK2, such as brain, kidneys, and lungs. However, few have explored its role in the liver that has a low LRRK2 expression. In inflammatory responses, classic acute inflammatory cytokines are significantly increased in the liver, brain, and peripheral circulation when hepatic encephalopathy occurs. Studies have documented significant increases in IL-1β, IL-6 and TNF-α in the brain and plasma in the TAA-induced rat ALF/HE model [40] and marked elevation of pro-inflammatory cytokines such as TNF-α, IL-1 and IL-6 in HE patients [41]. Likewise, the current study reported elevated levels of cytokines, such as IL-1β, IL-6, TNF- α, in the TAA-induced ALF and HE mouse models (a classical type A HE model), which was positively correlated with the enhanced liver inflammatory response. Notably, the *Lrrk*2^−/−^-HE mice reported the lowest survival rate, highest blood ammonia level, and severest inflammatory cell infiltration in the hepatic portal area; no significant difference in survival rate was evident between the *Lrrk2*^G2019S^-HE mice and WT-HE mice, suggesting that LRRK2 plays a protective role in the TAA-induced ALF and HE. However, it remains unraveled whether, despite its low baseline expression, LRRK2 enhances the hepatic inflammatory stimulation by regulating the activity of acute-phase inflammatory factors or otherwise. The pathogenicity of LRRK2 is mostly conferred by the gain of kinase function [42], which can be enhanced by G2019S mutation, the most common PD-related mutation [43]. In our study, the *Lrrk2*^G2019S^ mice reported a reduction in liver inflammation and mortality when compared with the *Lrrk2*^−/−^ mice, and no significant protective effect when compared with the WT mice, which was not reversed by G2019S mutation otherwise. A possible explanation may be that the LRRK2 overexpression in the *Lrrk2*^G2019S^ mice cannot completely replace the normal physiological functions of LRRK2.

Of interest, the current study documented different histopathological changes in the liver and brain, which may be attributed to the regional differences in LRRK2 expression in the TAA-HE mice. In the *Lrrk2*^−/−^-HE mice, LRRK2 deficiency significantly attenuated the TAA-induced injury to different brain regions, including the PFC and the dorsal STR that regulate motor function, the ventral STR that involves self-reward, and the HIP CA3 and dentate gyrus (DG) regions that modulate learning and memory. The available literature evidences that LRRK2-mediated immunoreactivity can degrade the myelin sheaths [44] and that small myelinated nerve fibers are crucial for maintaining a balance between excitation and inhibition in the brain hemispheres [45]. In the current study, the *Lrrk2*^G2019S^-HE mice reported a significant degeneration of myelin sheaths in the STR, especially the small myelinated nerve fibers, in which the mice, under the stressful circumstances, displayed motor coordination dysfunction in the challenging rotarod test. The above results suggest that the difference in LRRK2 backgrounds in HE patients may indicate a clinical translational significance in elucidating the pathophysiological mechanisms of various symptoms.

Noteworthily, the current study also revealed a LRRK2 imbalance-induced synaptic structural impairment in the TAA environment. In the available literature, controversies remain regarding the role of LRRK2 for synaptic vesicle transmission. Some previous studies have showed that LRRK2 overexpression can decrease the release of neurotransmitters of dopaminergic neurons, which is partly related to the LRRK2 overexpression-induced abnormal protein deposition in the synaptic membrane and vesicle membrane structure, thus impairing vesicle transport [46]. Other studies, however, have found that LRRK2 knockout impinges clathrin-mediated neurotransmission and vesicle endocytosis [47]. In this study, the TAA intervention induced the structural abnormalities in the vesicles of asymmetric and symmetric synapses in the PFC. Of note, the *Lrrk2*^−/−^ mice showed more typical vesicle structural abnormalities both at baseline and after the TAA injection, especially in asymmetric synapses. The results seem to indicate that the TAA-induced brain damage may aggravate the injury to vesicle endocytosis from LRRK2 knockout, resulting in synaptic transmission dysfunction, motor impairment, and lassitude.

Given the multiple functions of brain resident immune cells, microglia form the frontline defense of the innate immune system. Findings regarding the association between microglia and LRRK2 remain controversial however. Some studies have found that LRRK2 overexpression activates microglia [48, 49] while others suggest that LRRK2 knockdown increases the microglial mobility, which may be attenuated by LRRK2 G2019S mutation via the inhibition of focal adhesion kinase [50]. The current study found that LRRK2 knockdown failed to markedly activate the microglia and that inflammatory factors increased in the brain of *Lrrk2*^−/−^-HE mice, which may be attributed to the compensatory mechanisms of LRRK1 [51]. In the *Lrrk2*^G2019S^ mice, microglia were activated before the TAA intervention and displayed an amoebic-like morphological change afterwards, indicating functional depletion, which partially downregulates the inflammatory factors. Therefore, despite the differences in baseline expression in various tissues and organs, LRRK2, under the external pathogenic stress, may exert distinct or even opposite regulatory effects on the pathophysiological responses in systemic inflammatory diseases in order to protect the organs.

Autophagy, especially lysophagy, is most intensively studied in LRRK2-regulated inflammation, but the role of LRRK2 in different diseases remains controversial. Some studies have reported that LRRK2 overexpression decreases autophagy in the BMDCs from *Lrrk2* Tg mice that were exposed to an autophagy-inducing microbe *M. leprae* [52] and that the most common LRRK2 mutation, LRRK2-G2019S, significantly reduces the transport of autophagosome in neurons in a kinase-dependent manner [53], indicating that LRRK2 inhibits autophagy. Other studies, however, argue that the loss of LRRK2 also leads to dysfunction of the autophagic-lysosomal pathway [54]. Still others suggest that both the enhancement and loss of LRRK2 expression can affect the smooth progression of the whole process of autophagy [55], indicating that the expression and activity of LRRK2 must be appropriately moderated in order to regulate the normal autophagy pathway. The current study demonstrated an acute inflammatory response in the liver and brain of the TAA-HE mice, which was more serious in the former than in the latter, and an aggravated hepatic inflammation and autophagic dysregulation by LRRK2 deletion, which significantly increased the mortality of the *Lrrk2*^*−/−*^ -HE mice. The results of TEM reported more serious lysosomal disruption, autophagosomal accumulation, and significant autolysosomal reduction in the *Lrrk2*^−/−^-HE mice than in either the *Lrrk2*^G2019S^ -HE mice or the WT -HE mice. Western blotting revealed increased expression of classical protein markers of autophagy P62 and LC3B in TAA-induced ALF and HE mice, suggesting impaired autophagy flow [56]. Meanwhile, the expressions of LAMP1, LAMP2 and pro-Cath-D increased but mature Cath-D decreased in the ALF and HE mice, indicating aberrant functions of lysosomes and impaired autophagic-lysosomal pathway. Collectively, the above results suggest that the inhibition of autophagy flow impairs its protection against acute inflammation in the liver and that the knockout of LRRK2 can result in a severe blockage of the autophagic-lysosomal pathway.

Rab GTPases, as direct physiological substrates of LRRK2, are required for regulating autophagy [16]. LRRK2 has been documented to interact directly with several Rab GTPases, such as Rab5, Rab7, Rab8, and Rab10 [57]. In this study, we detected in lysosomes the expressions of Rab5, Rab7, Rab8, Rab10, Rab29, and LRRK2, and the localization of Rab10 and LRRK2. The results found no significant difference in the expressions of Rab5, Rab7, Rab8 and Rab29 among the groups after the TAA intervention. However, the expression of Rab10 increased and its phosphorylation decreased in the *Lrrk2*^−/−^-HE mice, suggesting that in the TAA-HE mice, LRRK2 may interfere with lysosomal homeostasis by regulating Rab10 phosphorylation, thereby affecting autophagy.

The results in this study reveal critical roles of LRRK2 in TAA-induced liver inflammation and autophagy regulation. LRRK2 is known to regulate inflammatory progression through multiple signaling pathways, including MAPK, NF-κB, and NFAT signaling pathways. In the pathogenesis of TAA-induced ALF/HE, the signaling pathway for LRRK2-regulated inflammation and the role of LRRK2 in the metabolism of blood ammonia remain unilluminated and warrant further explorations. Currently, LRRK2-G2019S mutation is one of the most common mutations in PD and inhibitors targeting LRRK2 are in the clinical trials in the PD treatment [58]. However, whether LRRK2 inhibitors affect the normal physiology of peripheral tissues, especially the liver, awaits further elucidation, despite that LRRK2 kinase inhibitors can effectively improve the clinical symptoms of PD patients by inhibiting kinase activity. So far, many studies have demonstrated that LRRK2 imbalance can seriously impact the physiological functions of kidneys and lungs [59]. However, few reports are available to elucidate the effect of LRRK2 on liver diseases, which is the original intention of this study.

In conclusion, to our knowledge, we demonstrate for the first time that LRRK2 deficiency may exacerbate TAA-induced ALF and HE in mice, and explore the involvement of LRRK2 in the inflammation and autophagic-lysosomal pathway in a TAA-induced HE model. Given the important role of LRRK2 in regulating TAA-induced liver and brain inflammatory responses in acute HE mice, sufficient care should be taken in evaluating the toxicities and indications of treatments targeting LRRK2.

### Supplementary Information

Supplementary Material 1.

## Data Availability

Not applicable.
