# Supplementary material for: Deficiency of leucine-rich repeat kinase 2 aggravates thioacetamide-induced acute liver failure and hepatic encephalopathy in mice
Source: J Neuroinflammation. 2024 May 9;21:123. doi: 10.1186/s12974-024-03125-4 (PMC11084037; doi:10.1186/s12974-024-03125-4)
Supplement: Supplementary file 1 — Supplementary Material 1. [file 12974_2024_3125_MOESM1_ESM.docx]

**Deficit of leucine-rich repeat kinase 2 aggravates thioacetamide-induced acute liver failure and hepatic encephalopathy in mice**

Dan Li^2,6,†^*, Shu-fang Yu^2,†^, Lin Lin^1,3,4†^, Jie-ru Guo^2†^, Si-mei Huang^2^, Xi-lin Wu^1,3,5^, Han-lin You^1,3^, Xiao-juan Cheng^1,3^, Qiu-yang Zhang^1^, Yu-qi Zeng^1,3^ and Xiao-dong Pan^1,3,4,5^*

**Supplemental Table. 1 Primer sequence of RT qPCR**

| **Gene** | **Primer sequences (5’ to 3’)** | |
| --- | --- | --- |
| GAPDH | Forward | CAGTGGCAAAGTGGAGATTGTTG |
|  | Reverse | CTCGCTCCTGGAAGATGGTGAT |
| IL-1β | Forward | TTCAGGCAGGCAGTATCACTC |
|  | Reverse | GAAGGTCCACGGGAAAGACAC |
| IL-4 | Forward | GAACGAGGTCACAGGAGAAGG |
|  | Reverse | AATATGCGAAGCACCTTGGAA |
| IL-6 | Forward | CTGCAAGAGACTTCCATCCAG |
|  | Reverse | AGTGGTATAGACAGGTCTGTTGG |
| IL-8 | Forward | TGTTGAGCATGAAAAGCCTCTAT |
|  | Reverse | AGGTCTCCCGAATTGGAAAGG |
| IL-10 | Forward | CTTACTGACTGGCATGAGGATCA |
|  | Reverse | GCAGCTCTAGGAGCATGTGG |
| CD68 | Forward  Reverse  Forward | TGTCTGATCTTGCTAGGACCG  TGTCTGATCTTGCTAGGACCG  GCAGCTCTAGGAGCATGTGG |
| CD86 | Reverse | TTGAGCCTTTGTAAATGGGCA |
| CD206 | Forward | CTCTGTTCAGCTATTGGACGC |
|  | Reverse | CGGAATTTCTGGGATTCAGCTTC |
| IFN-γ | Forward | CTTGGCAATACTCATGAATGCA |
|  | Reverse | CTTGAAAGACAATCAGGCCATC |
| TNF-α | Forward | CAGGCGGTGCCTATGTCTC |
|  | Reverse | CGATCACCCCGAAGTTCAGTAG |
| iNOS | Forward | CCTCCTCGTTCAGCTCACCT |
|  | Reverse | CAATCCACAACTCGCTCCAA |
| CCL7 | Forward | GCTGCTTTCAGCATCCAAGTG |
|  | Reverse | CCAGGGACACCGACTACTG |
| Arg1  LRRK2  CD45 | Forward | CAATGAAGAGCTGGCTGGTG |
|  | Reverse  Forward  Reverse  Forward  Reverse | GGCCAGAGATGCTTCCAACT  TCCTACTCTGGGAGGGTGAA  TTGTGAATGGTGCGGATAAC  GTTTTCGCTACATGACTGCACA  AGGTTGTCCAACTGACATCTTTC |

**Supplemental Figure 1**





**Supplemental Figure 2**

**
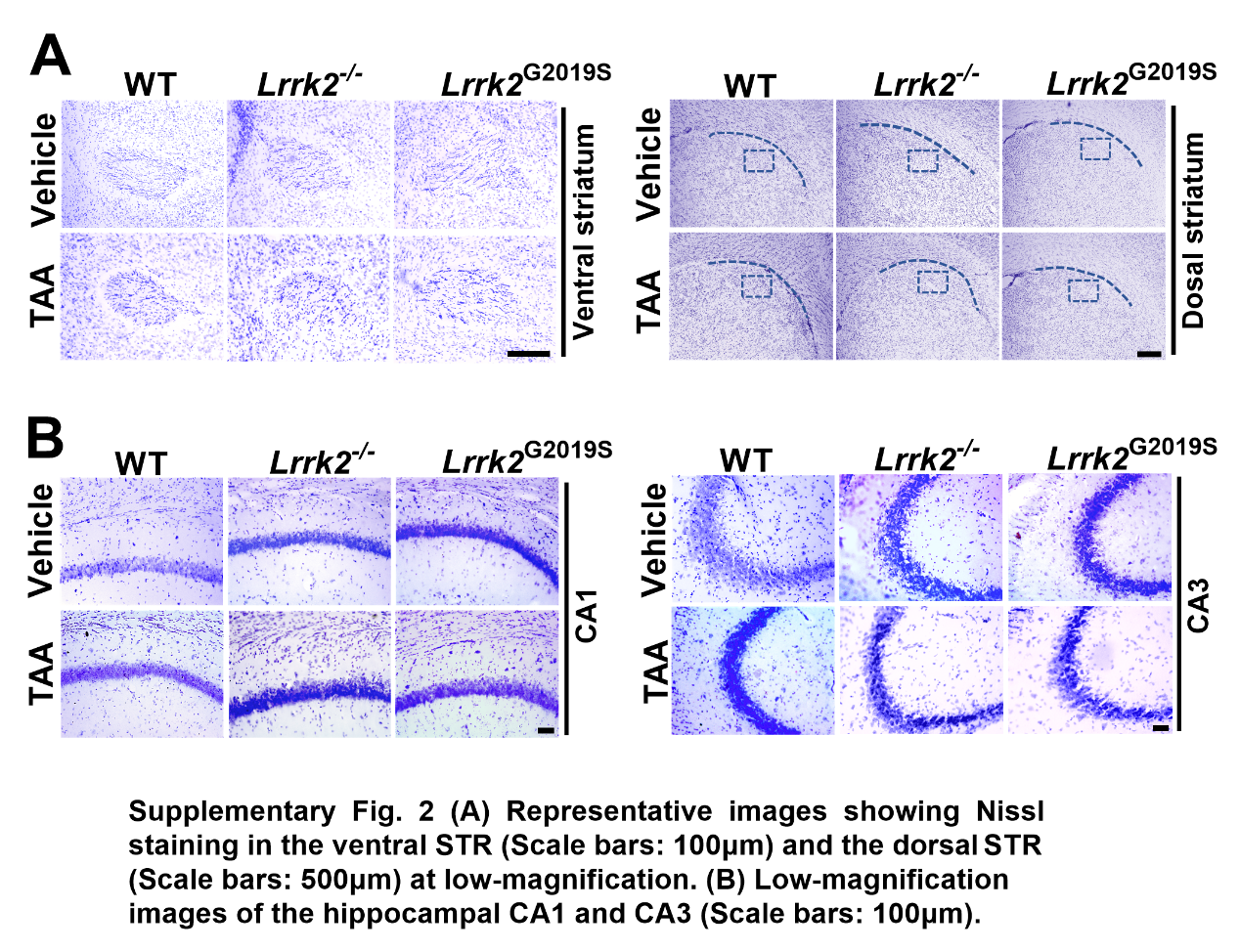
**

**Supplemental Figure 3**


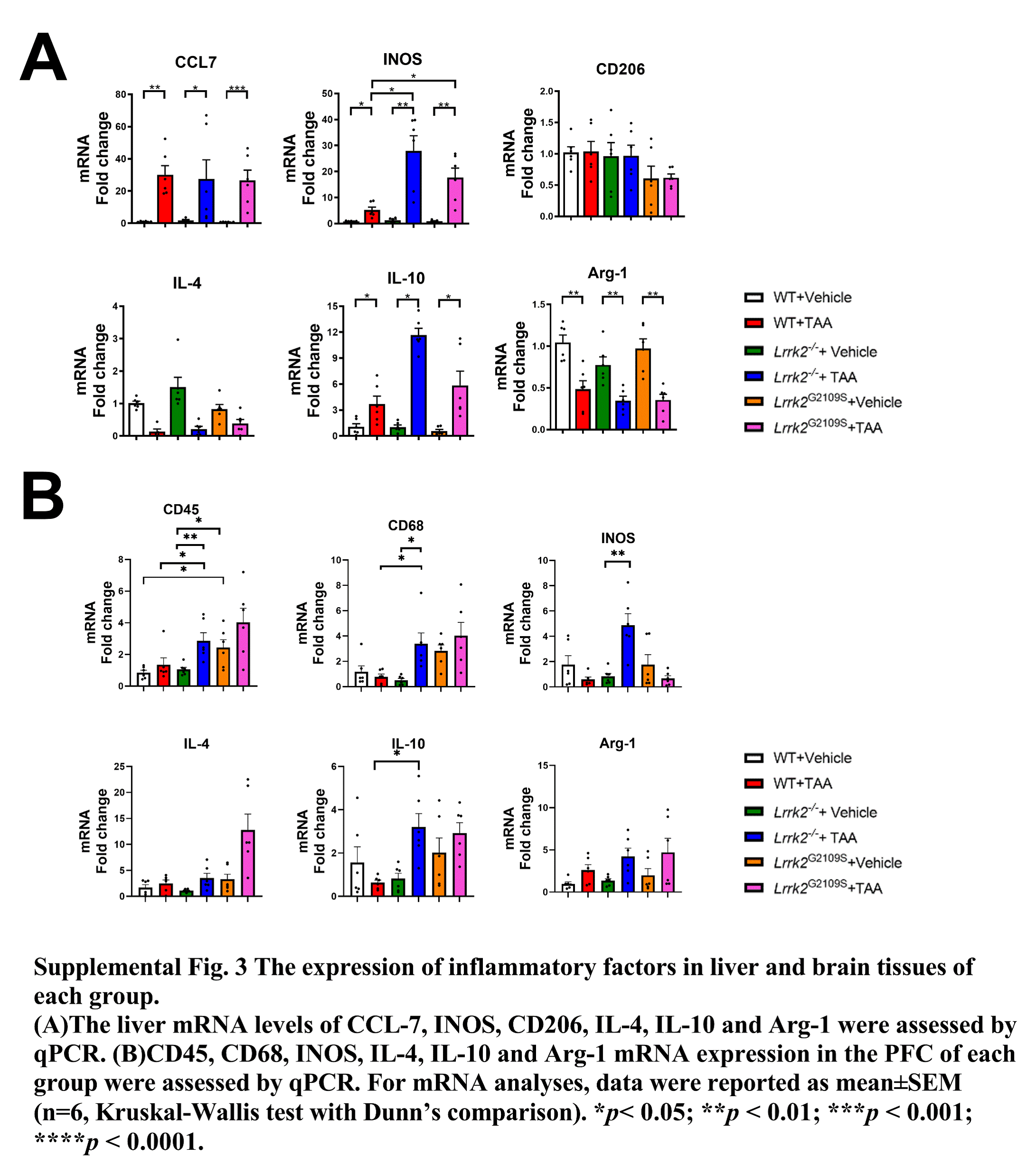


**Supplemental Figure 4**

**

**

**Supplemental Figure 5**





**Supplemental Table. 2**

**Body weight changes in WT+Vehicle group（g）**

| Ear tag | 0h | 24h after the first injection | 48h after the first injection |
| --- | --- | --- | --- |
| A614 | 22.4 | 22.4 | 21.9 |
| A619 | 25.5 | 25.7 | 25.7 |
| A693 | 23.2 | 23.0 | 23.1 |
| A577 | 21.2 | 21.1 | 21.3 |
| A695 | 17.3 | 17.2 | 17.8 |
| A687 | 25.0 | 25.2 | 24.7 |
| A688 | 23.0 | 23.4 | 22.8 |
| A696 | 25.3 | 25.4 | 26.0 |
| A699 | 27.5 | 27.7 | 26.4 |
| A616 | 19.4 | 19.4 | 20.8 |
| A462 | 28.2 | 28.6 | 27.7 |
| A473 | 29.0 | 28.9 | 28.7 |
| A702 | 23.4 | 23.0 | 22.7 |
| A762 | 26.0 | 25.8 | 25.2 |
| A985 | 21.6 | 21.3 | 21.0 |
| A682 | 21.7 | 22.1 | 22.6 |
| A450 | 17.5 | 18.5 | 19.6 |
| A687 | 21.1 | 20.6 | 20.6 |
| A35 | 25.2 | 25.2 | 25.7 |
| A34 | 25.4 | 25.9 | 25.6 |
| A853 | 27.5 | 27.3 | 27.5 |
| A856 | 28.4 | 28.5 | 28.6 |
| A827 | 25.5 | 25.4 | 23.3 |
| A852 | 24.2 | 24.6 | 24.8 |
| A76 | 32.2 | 32.2 | 32.8 |
| A77 | 30.0 | 29.8 | 29.8 |
| A78 | 25.3 | 24.9 | 25.0 |
| A79 | 23.0 | 22.7 | 22.3 |
| A80 | 26.8 | 26.3 | 26.6 |
| A81 | 27.1 | 27.0 | 26.5 |
| A421 | 22.2 | 22.0 | 21.9 |
| A424 | 24.6 | 24.6 | 24.9 |
| A425 | 26.1 | 25.5 | 25.8 |
| A427 | 22.6 | 22.9 | 23 |
| A428 | 24.9 | 25.2 | 24.9 |
| A429 | 21.8 | 22.0 | 21.4 |
| A370 | 24.5 | 24.7 | 24.9 |
| A371 | 23.7 | 23.8 | 23.5 |
| A372 | 21.7 | 21.0 | 21.4 |
| A373 | 25.4 | 25.4 | 25.4 |
| A374 | 27.3 | 27.4 | 25.5 |
| A375 | 26.1 | 26.0 | 25.3 |
| A376 | 24.4 | 24.0 | 23.8 |
| A377 | 23.9 | 24.1 | 24.3 |
| A241 | 25.0 | 25.5 | 25.2 |
| A245 | 23.2 | 23.5 | 22.6 |

**Body weight changes in WT+TAA group（g）**

| Ear tag | 0h | 24h after the first injection | 48h after the first injection |
| --- | --- | --- | --- |
| B615 | 21.2 | 20.4 | Die |
| B620 | 26.8 | 23.8 | 26.3 |
| B689 | 24.8 | 24.2 | 23.0 |
| B690 | 22.4 | 21.5 | 21.0 |
| B691 | 24.2 | 24.0 | Die |
| B692 | 19.8 | 17.3 | 18.6 |
| B212 | 21.6 | 20.8 | 19.8 |
| B472 | 25.2 | 25.1 | 25.0 |
| B460 | 29.4 | 26.0 | Die |
| B461 | 31.7 | 27.8 | Die |
| B462 | 30.6 | Die | Die |
| B464 | 28.8 | Die | Die |
| B465 | 28.8 | 25.7 | 24.1 |
| B702 | 25.8 | 25.5 | 25.0 |
| B703 | 26.5 | Die | Die |
| B704 | 32.3 | 27.5 | Die |
| B891 | 21.4 | 20.2 | 19.4 |
| B892 | 22.0 | 21.2 | 20.4 |
| B251 | 20.5 | 20.4 | 18.5 |
| B252 | 21.4 | 21.2 | Die |
| B254 | 22.9 | 23.4 | 21.7 |
| B473 | 30.9 | Die | Die |
| B762 | 21.9 | Die | Die |
| B985 | 24.1 | Die | Die |
| B888 | 25.6 | Die | Die |
| B889 | 22.4 | 22.0 | Die |
| B894 | 23.7 | Die | Die |
| B895 | 22.1 | Die | Die |
| B896 | 21.7 | Die | Die |
| B552 | 26.5 | 25.7 | 25.1 |
| B553 | 22.7 | 22,0 | 21.6 |
| B543 | 27.6 | 27.0 | 26.5 |
| B554 | 24.8 | 24.4 | Die |
| B544 | 16.4 | 16.2 | 16.1 |
| B545 | 19.6 | 19.1 | 18.5 |
| B548 | 15.6 | Die | Die |
| B88 | 23.7 | 22.4 | 21.5 |
| B89 | 22.0 | 21.0 | Die |
| B90 | 21.9 | 21.2 | Die |
| B677 | 23.7 | 22.4 | 22.1 |
| B678 | 26.0 | 24.8 | 24.9 |
| B679 | 23.6 | 22.6 | 21.6 |
| B680 | 28.3 | Die | Die |
| B681 | 30.0 | Die | Die |
| B683 | 23.1 | 22.0 | Die |
| B684 | 20.4 | 19.0 | 18.4 |
| B688 | 22.8 | 21.0 | Die |
| B37 | 27.1 | 24.9 | 23.3 |
| B38 | 27.5 | 25.0 | 23.6 |
| B39 | 24.4 | 22.6 | Die |

**Body weight changes in** ***Lrrk2*^-/-^ +Vehicle group ( g )**

| Ear tag | 0h | 24h after the first injection | 48h after the first injection |
| --- | --- | --- | --- |
| C249 | 30.3 | 30.4 | 30.5 |
| C229 | 26.0 | 25.7 | 25.7 |
| C417 | 27.0 | 27.2 | 27.2 |
| C701 | 28.6 | 28.2 | 28.3 |
| C844 | 29.6 | 29.7 | 29.6 |
| C854 | 26.1 | 25.5 | 26.5 |
| C858 | 27.8 | 27.7 | 27.6 |
| C859 | 25.4 | 25.1 | 24.8 |
| C659 | 22.4 | 21.8 | 21.4 |
| C634 | 25.6 | 25.3 | 24.9 |
| C712 | 22.4 | 22.4 | 21.1 |
| C435 | 23.4 | 23.5 | 23.9 |
| C632 | 29.3 | 29.6 | 29.5 |
| C65 | 18.9 | 18.6 | 18.6 |
| C692 | 24.4. | 24.7 | 24.5 |
| C651 | 24.2 | 24.6 | 24.7 |
| C711 | 30.2 | 30.6 | 30.7 |
| C200 | 29.0 | 28.1 | 28.5 |
| C475 | 27.4 | 27.4 | 27.6 |
| C465 | 26.7 | 26.0 | 25.8 |
| C467 | 25.5 | 24.9 | 24.9 |
| C468 | 26.3 | 26.3 | 26.4 |
| C469 | 27.7 | 27.8 | 27.9 |
| C505 | 23.4 | 23.6 | 23.9 |
| C506 | 23.7 | 24.1 | 24.3 |
| C507 | 22.0 | 21.8 | 21.6 |
| C508 | 25.3 | 25.2 | 25.1 |
| C509 | 24.8 | 24.9 | 24.9 |
| C510 | 26.0 | 26.3 | 26.5 |
| C511 | 23.6 | 22.9 | 22.7 |
| C512 | 23.7 | 23.8 | 23.9 |
| C513 | 27.1 | 27.2 | 27.0 |
| C145 | 28.2 | 28.5 | 28.6 |
| C147 | 26.5 | 26.3 | 26.3 |
| C148 | 23.0 | 23.4 | 23.5 |
| C149 | 24.8 | 24.8 | 24.9 |
| C381 | 21.9 | 21.8 | 21.5 |
| C385 | 26.6 | 26.0 | 26.5 |
| C388 | 25.9 | 25.5 | 25.0 |
| C389 | 26.0 | 26.7 | 26.4 |
| C732 | 22.5 | 23.2 | 23.5 |
| C737 | 21.6 | 21.9 | 22.4 |
| C739 | 26.2 | 26.0 | 25.9 |
| C740 | 25.0 | 25.4 | 25.3 |
| C741 | 27.1 | 27.1 | 27.2 |

**Body weight changes in** ***Lrrk2*^-/-^ +TAA group（g）**

| Ear tag | 0h | 24h after the first injection | 48h after the first injection |
| --- | --- | --- | --- |
| D91 | 19.7 | 19.5 | 18.7 |
| D525 | 25.6 | Die | Die |
| D962 | 27.4 | 26.1 | 25.0 |
| D677 | 26.7 | Die | Die |
| D678 | 28.7 | 26.6 | Die |
| D679 | 26.2 | Die | Die |
| D680 | 27.1 | Die | Die |
| D681 | 26.2 | Die | Die |
| D683 | 30.0 | Die | Die |
| D684 | 31.1 | Die | Die |
| D686 | 28.1 | 27.1 | 26.3 |
| D727 | 31.2 | 30.1 | Die |
| D899 | 24.1 | 23.4 | 23.9 |
| D418 | 28.9 | 27.3 | 25.0 |
| D66 | 24.1 | Die | Die |
| D67 | 25.2 | Die | Die |
| D68 | 18.6 | Die | Die |
| D849 | 25.8 | Die | Die |
| D249 | 23.3 | Die | Die |
| D890 | 27.8 | 26.7 | 26.0 |
| D852 | 25.2 | Die | Die |
| D405 | 31.7 | 29.4 | 29.1 |
| D865 | 32.4 | 30.5 | 30.2 |
| D493 | 27.8 | Die | Die |
| D492 | 27.0 | 24.9 | Die |
| D690 | 29.5 | Die | Die |
| D683 | 29.8 | Die | Die |
| D837 | 29.2 | 27.7 | Die |
| D125 | 27.1 | 25.4 | 25.7 |
| D393 | 28.8 | Die | Die |
| D395 | 27.3 | 25.9 | Die |
| D390 | 28.0 | 26.4 | Die |
| D176 | 26.4 | Die | Die |
| D389 | 26.4 | Die | Die |
| D386 | 29.1 | Die | Die |
| D399 | 26.5 | 25.3 | Die |
| D139 | 29.7 | 27.3 | 26.5 |
| D633 | 27.2 | 26.9 | Die |
| D368 | 29.7 | Die | Die |
| D700 | 27.3 | 26.8 | 25.6 |
| D307 | 30.2 | Die | Die |
| D460 | 29.2 | Die | Die |
| D459 | 29.4 | Die | Die |
| D452 | 27.6 | Die | Die |
| D305 | 28.3 | Die | Die |
| D456 | 26.3 | Die | Die |

**Body weight changes in** ***Lrrk2*^G2019S^*+*Vehicle group ( g )**

| Ear tag | 0h | 24h after the first injection | 48h after the first injection |
| --- | --- | --- | --- |
| E204 | 30.0 | 30.3 | 30.0 |
| E206 | 29.5 | 29.7 | 29.9 |
| E301 | 28.0 | 28.1 | 28.1 |
| E320 | 26.4 | 26.2 | 26.0 |
| E321 | 22.0 | 22.1 | 22.1 |
| E807 | 21.9 | 22.4 | 22.8 |
| E810 | 19.9 | 20.3 | 20.3 |
| E811 | 21.0 | 21.2 | 21.3 |
| E273 | 24.7 | 24.7 | 24.5 |
| E274 | 24.0 | 24.4 | 24.1 |
| E833 | 23.0 | 22.6 | 22.0 |
| E945 | 26.6 | 27.2 | 26.7 |
| E678 | 19.7 | 19.5 | 19.7 |
| E760 | 21.4 | 21.9 | 22.1 |
| E248 | 24.8 | 25.2 | 25.5 |
| E229 | 26.0 | 26.2 | 27.5 |
| E108 | 28.5 | 28.6 | 28.2 |
| E73 | 22.9 | 23.8 | 23.8 |
| E52 | 23.5 | 23.2 | 23.0 |
| E53 | 25.0 | 25.0 | 24.8 |
| E54 | 27.5 | 27.6 | 27.9 |
| E55 | 26.2 | 26.6 | 26.3 |
| E56 | 26.9 | 26.9 | 26.8 |
| E57 | 23.3 | 23.4 | 23.9 |
| E58 | 26.7 | 26.4 | 26.4 |
| E59 | 28.3 | 28.3 | 28.3 |
| E666 | 22.5 | 22.7 | 22.8 |
| E667 | 21.0 | 21.4 | 21.4 |
| E668 | 24.3 | 24.0 | 23.7 |
| E669 | 26.7 | 26.4 | 26.0 |
| E670 | 26.2 | 26.0 | 26.3 |
| E671 | 25.6 | 25.6 | 25.6 |
| E672 | 24.4 | 24.6 | 24.8 |
| E673 | 22.8 | 22.4 | 22.7 |
| E674 | 24.0 | 24.0 | 24.3 |
| E675 | 27.7 | 27.4 | 27.4 |
| E676 | 25.9 | 26.3 | 26.5 |
| E677 | 25.1 | 24.7 | 24.7 |
| E678 | 26.6 | 26.6 | 26.4 |
| E679 | 24.8 | 24.0 | 24.6 |
| E697 | 22.0 | 22.2 | 22.0 |
| E698 | 28.0 | 27.8 | 27.5 |
| E783 | 26.3 | 26.0 | 25.9 |
| E784 | 24.4 | 24.6 | 24.6 |
| E785 | 25.6 | 25.6 | 25.7 |

**Body weight changes in** ***Lrrk2*^G2019S^*+*TAA group ( g )**

| Ear tag | 0h | 24h after the first injection | 48h after the first injection |
| --- | --- | --- | --- |
| F611 | 20.6 | 20.0 | 19.8 |
| F612 | 22.9 | 20.8 | 20.7 |
| F613 | 21.4 | 19.9 | 19.7 |
| F622 | 25.5 | Die | Die |
| F623 | 26.2 | 25.6 | 25.3 |
| F618 | 26.2 | 24.1 | Die |
| F319 | 26.1 | 26.4 | 24.6 |
| F812 | 29.1 | 26.8 | Die |
| F813 | 27.9 | 26.0 | 24.7 |
| F814 | 26.4 | Die | Die |
| F815 | 26.2 | 24.7 | 24.0 |
| F816 | 29.0 | Die | Die |
| F429 | 23.2 | 22.5 | 21.3 |
| F201 | 29.0 | 28.6 | 26.2 |
| F203 | 25.8 | 25.4 | 23.0 |
| F525 | 22.5 | Die | Die |
| F164 | 22.9 | 21.9 | 20.1 |
| F711 | 19.1 | 18.2 | 16.8 |
| F900 | 23.4 | 23.1 | 21.9 |
| F896 | 22.7 | 21.7 | 21.0 |
| F804 | 21.5 | Die | Die |
| F817 | 25.1 | 23.5 | 22.9 |
| F818 | 27.7 | Die | Die |
| F621 | 29.2 | Die | Die |
| F822 | 31.0 | 28.2 | Die |
| F952 | 26.8 | 26.7 | Die |
| F730 | 24.2 | 28.1 | 25.5 |
| F759 | 25.8 | 23.4 | 22.5 |
| F46 | 28.1 | Die | Die |
| F89 | 21.9 | 21.1 | Die |
| F45 | 21.3 | 22.7 | Die |
| F797 | 25.0 | Die | Die |
| F790 | 29.6 | Die | Die |
| F322 | 26.6 | Die | Die |
| F40 | 26.7 | 24.2 | 23.0 |
| F100 | 23.6 | 22.2 | 21.1 |
| F313 | 22.8 | 22.5 | 20.2 |
| F314 | 23.1 | Die | Die |
| F159 | 19.9 | 20.2 | 20.1 |
| F74 | 23.5 | Die | Die |
| F170 | 23.8 | 22.6 | 22.2 |
| F195 | 17.7 | Die | Die |
| F196 | 21.2 | Die | Die |
| F197 | 23.5 | 22.3 | 21.8 |
| F198 | 24.4 | Die | Die |
| F199 | 25.2 | Die | Die |
| F207 | 22.0 | 21.0 | 20.7 |
| F209 | 25.7 | Die | Die |
| F300 | 24.1 | Die | Die |
| F308 | 26.7 | Die | Die |

**Supplemental Table. 3 List of antibodies used in WB**

|  | Supplier | Cat No. |
| --- | --- | --- |
| LRRK2 | Abcam | ab133474 |
| LRRK2 (phosphor-Ser935) | Abcam | ab133450 |
| CD68 | Santa Cruz | sc-20060 |
| CD45 | Abcam | ab10558 |
| LAMP1 | Thermo Fisher | 14-1071-82 |
| LAMP2 | Abcam | ab13524 |
| Cathepsin D | Abcam | ab75852 |
| Rab5 | CST | 3547S |
| Rab7 | Abcam | ab137029 |
| Rab8a | Abcam | ab188574 |
| Rab8a (phosphor-Thr72) | Abcam | ab230260 |
| Rab10 | Abcam | ab104859 |
| Rab10 (phosphor-Thr73) | Abcam | ab230261 |
| Rab29 | Sigma-Aldrich | SAB4503507 |
| LC3B | Sigma-Aldrich | L7543 |
| SQSTM1/p62 | CST | 88588S |
| Beclin-1 | CST | 3495T |
| GAPDH | CST | 2118S |
